# Health Coaching and Its Impact in the Remote Management of Patients With Type 2 Diabetes Mellitus: Scoping Review of the Literature

**DOI:** 10.2196/60703

**Published:** 2025-04-09

**Authors:** Jun Jie Benjamin Seng, Hosea Nyanavoli, Glenn Moses Decruz, Yu Heng Kwan, Lian Leng Low

**Affiliations:** 1 Department of Medicine MOH Holdings Singapore Singapore; 2 SingHealth Polyclinics Singapore Singapore; 3 Family Medicine Academic Clinical Program SingHealth Duke-NUS Academic Medical Centre Singapore Singapore; 4 Yong Loo Lin School of Medicine National University of Singapore Singapore Singapore; 5 Department of Rheumatology and Immunology Singapore General Hospital Singapore Singapore; 6 Program in Health Services and Systems Research Duke-NUS Medical School Singapore Singapore; 7 Research and Translational Innovation SingHealth Community Hospitals Singapore Singapore; 8 Department of Family Medicine and Continuing Care Singapore General Hospital Singapore Singapore

**Keywords:** diabetes mellitus, type 2, remote consultation, telenursing, education, health coaching, scoping review, telemonitoring, PRISMA

## Abstract

**Background:**

Health coaching refers to the practice of health education and promotion to drive goal-directed behavioral changes and improve an individual’s well-being. Remote patient monitoring systems, which employ health coaching interventions, have been gaining interest and may aid in the management of patients with type 2 diabetes mellitus (T2DM).

**Objective:**

This scoping review aims to summarize the impact of health coaching in the remote monitoring of patients with T2DM.

**Methods:**

A scoping review was performed in MEDLINE, Embase, CINAHL, PsychInfo, and Web of Science up to September 2024 and was reported using the PRISMA-ScR (Preferred Reporting Items for Systematic Reviews and Meta-Analyses extension for Scoping Reviews) checklist. The initial abstract screening, full-text review, and data extraction were performed by 2 independent reviewers. Studies that evaluated the impact of health coaching on the remote management of patients with T2DM were included. Outcomes evaluated were grouped into clinical, humanistic, psychiatric, behavioral, knowledge, and economic domains. A narrative review was performed for the impact of health coaching on the remote management of patients with T2DM.

**Results:**

Among 168,888 citations identified, 104 studies were included. Majority of the studies were conducted in North America (56/104, 53.8%) and Asia (30/104, 28.8%). Approximately half of the studies (48/104, 46.2%) were conducted in primary health care settings, and one-third of the studies (37/104, 35.6%) employed nurses as health coaches. Phone consultations were the most common modality of remote monitoring (45/104, 43.3%). The follow-up duration of most studies (64/104, 61.5%) was less than 1 year. Regarding clinical outcomes, majority of the studies (68/92, 73%) showed improvements in diabetes-related parameters, but there was no improvement in blood pressure (21/32, 66%) or hyperlipidemia control (19/32, 59%). For humanistic outcomes, health coaching was associated with higher satisfaction with diabetes-related care (10/11, 91%), but there was no improvement in quality of life (12/20, 60%). Regarding psychiatric outcomes, there was no association with improvement in depressive (8/14, 57%) or anxiety symptoms (4/5, 80%). For behavioral outcomes, most studies (12/19, 63%) showed improvement in diabetes-related self-efficacy. For knowledge outcomes, evidence was mixed, with half of the studies (5/9, 56%) showing improvement in diabetes-related knowledge. For economic outcomes, majority of the studies (8/11, 73%) did not show a reduction in health care use.

**Conclusions:**

Health coaching was associated with improved diabetes control and self-management among patients with T2DM on remote monitoring. Its role appears limited in improving health care use, lipid parameters, and quality of life; however, this may have been confounded by the short duration of follow-up in the studies. More studies are required to identify the optimal modality and duration of digital health coaching for patients with T2DM.

## Introduction

Type 2 diabetes mellitus (T2DM) is a global health problem, which affects over 463 million people worldwide and has been projected to affect 700 million people by 2045 [1]. Direct and indirect costs arising from diabetes care and its complications led to health care expenditure of US $727 billion in 2017, and this is expected to rise to US $825 billion by 2045.

The recent COVID-19 pandemic resulted in the implementation of unprecedented containment measures, such as social distancing, lockdown of countries, and widespread use of personal protective equipment globally [2]. To mitigate excessive patient traffic in health care institutions while ensuring continuity of patient care during the pandemic, there was accelerated scale-up of remote patient monitoring and telehealth technologies and services for the provision of health services in many countries [3]. Of note, the use of teleconsultation increased over 50 times during the pandemic compared with the prepandemic period [4], and health care expenditure related to telehealth has been projected to increase to US $250 billion in the United States [5].

Through the use of digitally transmitted patient information via telephone, internet, wearable devices, or videoconferencing, remote patient monitoring has shown promising results in the early detection of disease complications and decompensation [6]. This in turn facilitates early implementation of interventions and patient education to improve patient self-management and outcomes [6]. For example, a review that evaluated the role of telemonitoring interventions among patients with chronic heart failure showed that these interventions reduced all-cause mortality and heart failure–related hospitalization compared to usual care [6]. Another review that assessed the role of home blood pressure telemonitoring showed marked improvement in blood pressure control in the intervention groups [7]. Specifically, among patients with T2DM, remote management of patients was shown to be superior to usual care in achieving a greater reduction in HbA_1c_ [8] and offers the benefits of shielding patients from health care environments with high concentrations of communicable diseases and saving on transportation time and costs for patients living in rural areas.

With mounting evidence supporting the role of lifestyle behaviors in chronic disease prevention and management, the delivery of health coaching via telemedicine and remote patient monitoring may aid in the management of patients with T2DM. Health coaching broadly refers to the practice of health education and promotion to facilitate health behavioral changes and improve health outcomes through interactions between a health care professional (coach) and an individual [9]. Trials that evaluated the role of health coaching in the remote monitoring of patients with T2DM have shown promising results. For example, the study by Bollyky et al [10] assessed the role of remote lifestyle coaching alongside access to diabetes educators and showed improved blood glucose control and greater weight loss among patients with T2DM. Another program that evaluated web-based health coaching for diabetes self-management and support found that patients who underwent the program had better medication adherence and exercise habits, and reduced depressive symptoms [11].

Existing reviews have examined outcomes associated with remote patient monitoring [6,7] in patients with heart failure or hypertension, or health coaching among the general population [12,13]. Other reviews that evaluated the impact of health coaching in the remote monitoring of patients were conducted for specific interventions, such as mobile Health (mHealth) [14], or in selected patient populations, such as elderly people [15] and pregnant women [16]. To the best of our knowledge, no review has been performed for patients with T2DM. Hence, the objective of this study was to evaluate and summarize the role and impact of health coaching on the remote management of patients with T2DM. A scoping review was adopted over a systematic review to allow for the mapping of the literature and the identification of potential knowledge gaps regarding the role of health coaching in the remote monitoring of patients with T2DM.

## Methods

### Protocol and Registration

The protocol of this scoping review has been registered on Open Science Framework (registration ID: x62zd), and the review has been reported in accordance with the PRISMA (Preferred Reporting Items for Systematic Reviews and Meta-Analyses) checklist.

### Information Sources and Search Strategy

A scoping review was conducted with searches in the MEDLINE, Embase, CINAHL, PsychInfo, and Web of Science databases. There was no restriction on the start date of the search to allow for comprehensive capture of literature, as conventional remote monitoring modalities, such as teleconsultation, are still used to date. Studies up to July 2023 were initially included. The search strategy encompassed key terms and Medical Subject Heading (MeSH) terms related to T2DM, health coaching, and remote management of patients, which were adapted from other systematic reviews conducted in other patient populations [17-21]. Discussions were performed with our institution’s librarian on the finalization of the search terms. The details of the full search strategy are presented in Multimedia Appendix 1. A subsequent update of the review was performed up to September 18, 2024, to ensure relevance of the scoping review. Details related to the search query from each database are included in Multimedia Appendix 2.

### Key Definitions

In this review, health coaching was defined as “the practice of health education and health promotion within a coaching context to enhance the well-being of individuals and to facilitate achievement of their health-related goals” [22]. The modality of health coaching interventions may include that delivered via telephone or the internet, in person, or through a combination of multiple delivery methods. Currently, there are no standardized definitions regarding the remote management of patients. For this review, the remote management of patients was defined as the use of digital technology to capture a patient’s health information in real-time and thereafter transmit it for evaluation by a health care professional or for self-management [23]. It includes obtaining patient health information via telephone consultation and videoconferencing, as well as the use of automated devices and communication networks for the transmission and delivery of health care services or information across different geographical locations [23,24].

### Eligibility Criteria and Selection Process

Full-text articles in the English language, which evaluated the role and impact of health coaching in the remote management of patients with T2DM, were included. Using the PICOS (patient, intervention, comparator, outcomes, and study) framework [25], the target population of interest included adult patients with T2DM (aged 18 years or older), and interventions included health coaching in the remote monitoring of patients. Comparator groups included patients who received usual care or other interventions. The outcomes of interest included clinical outcomes, such as diabetes and blood pressure control; humanistic outcomes, such as health-related quality of life; psychiatric outcomes, such as depressive and anxiety-related symptoms; behavioral outcomes, such as adherence to exercise and dietary modification; knowledge-related outcomes, such as diabetes-related knowledge; and economic outcomes, such as health care utilization and related costs. Study designs included randomized controlled trials, cross-sectional studies, cohort studies, observational studies, qualitative studies, and quasiexperimental and mixed methods studies. We excluded studies that evaluated only health coaching or the remote management of patients with T2DM and studies that included patients with type 1 diabetes mellitus or maturity-onset diabetes of the young. Case reports, series, study protocols, irrelevant systematic reviews, and meta-analyses were excluded. A detailed list of the inclusion and exclusion criteria is provided in Multimedia Appendix 3.

References and abstracts that were identified from the literature search were extracted to EndNote X9 software, where duplicate citations were removed. The screening of the titles, abstracts, and full texts of the retrieved citations was performed by 2 independent reviewers (JJBS and GMD) to identify relevant articles for inclusion in the review. A pilot screening test was performed for the first 200 citations to smoothen and ensure congruency in the screening process. The initial interrater agreement between JJBS and GMD was 92%. All disagreements during the screening process were discussed, and any unresolved disagreements were arbitrated by a third independent reviewer (HN). In addition, hand searching of references within the included studies was performed to identify other relevant studies.

### Data Collection Process and Data Items

A standardized Microsoft Excel form was used for the data collection process, and data were extracted independently by 2 reviewers. A pilot data extraction was performed for the first 20 citations to ensure accuracy of data extraction. The information collected from studies included the study title, publication year, sample size, characteristics of the patient population, details related to the type and duration of health coaching used, and modality of remote monitoring. In addition, information related to study outcomes was collected and grouped into 6 domains, namely clinical, behavioral, knowledge, humanistic, economic, and other domains [26]. Clinical outcomes included medical and medication-related outcomes, and humanistic outcomes included factors related to patients’ functional status and health-related quality of life. Economic outcomes included health care cost and utilization.

### Effect Measures and Synthesis Methods

A narrative review was performed for the impact of health coaching on the remote management of patients. The full details of the characteristics of the included studies are provided in Multimedia Appendix 4, and descriptive statistics were used to summarize the characteristics of the included studies. Continuous variables have been reported as mean (SD), and categorical variables have been reported as frequency (percentage). For studies with missing data, the corresponding authors were contacted for clarification. Information that could not be obtained after 2 email reminders were labeled as unavailable. Data imputation was not performed for this review.

To assess the suitability of meta-analyses for specific interventions in this review, the clinical and methodological heterogeneity of the included studies were examined by 2 independent reviewers (JJBS and GMD). Clinical heterogeneity refers to variation in the characteristics of the patient population, study intervention, or outcomes. In contrast, methodological heterogeneity refers to variation in the study design or risk of bias. Due to the expected heterogeneity of the included studies, meta-analyses were not performed.

## Results

### Study Characteristics

**Figure 1:**
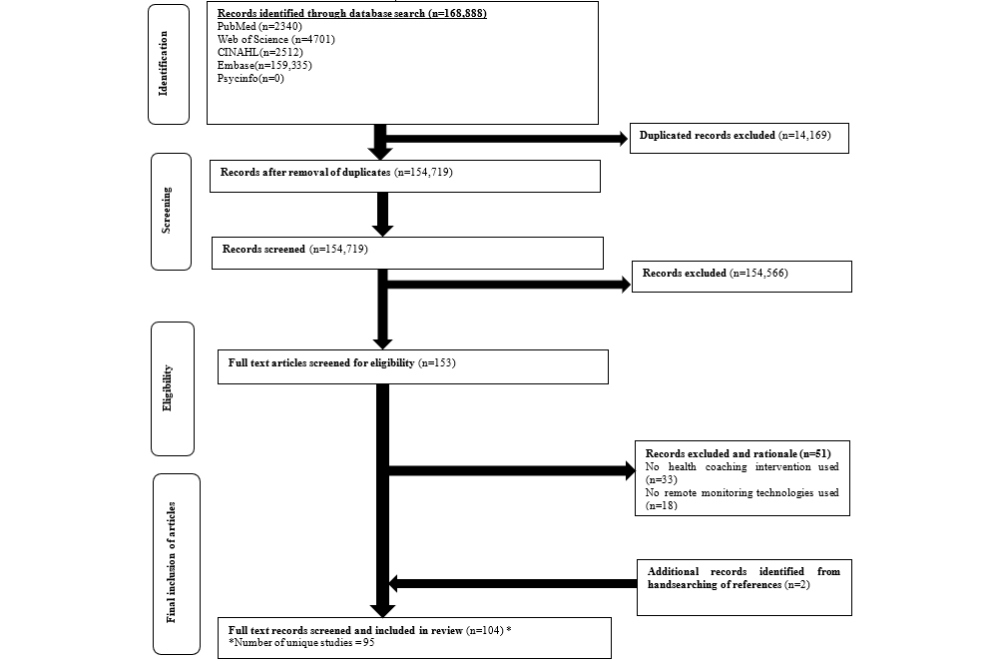
Flowchart of the inclusion of articles.

From the initial 168,888 citations, a total of 104 articles were included in this review, among which there were 95 unique studies (Figure 1). There was no deviation from the initial study protocol, except for a subsequent update to the review to ensure its relevance. The interrater reliability rate between the 2 independent reviewers was 93%. Table 1 provides a summary of the characteristics of the included studies. Majority of the studies (62/104, 59.6%) were conducted between 2011 and 2021. Most of the studies were conducted in North America (56/104, 53.8%), and the most common study design was randomized controlled trial (76/104, 73.1%). Most studies were conducted in the primary health care setting (48/104, 46.2%). The health coaches engaged in studies included nurses (37/104, 35.6%), multidisciplinary teams (19/104, 18.3%), diabetes educators (12/104, 11.5%), researchers (5/104, 4.8%), care coordinators (4/104, 3.8%), dieticians (4/104, 3.8%), pharmacists (4/104, 3.8%), medical doctors (2/104, 1.9%), and physiotherapists (2/104, 1.9%). On the other hand, the remote monitoring modalities used included phone consultation (45/104, 43.3%), automated devices/wearable devices (18/104, 17.3%), teleconsultation (17/104, 16.3%), digital applications/websites (17/104, 16.3%), and self-reported questionnaires (7/104, 6.7%). Majority of the studies (64/104, 61.5%) had a follow-up period of 1 year or less. Complete details of the included studies are provided in Multimedia Appendix 4.

**Table 1:** Characteristics of the included studies.

Characteristic				Value (N=104), n (%)
**Year of the study**				
	2000 or earlier		3 (2.9)	
	2001-2010		14 (13.5)	
	2011-2021		62 (59.6)	
	2022-2024		25 (24.0)	
**Continent of the study**				
	North America		56 (53.8)	
	Asia		30 (28.8)	
	Europe		15 (14.4)	
	South America		2 (1.9)	
	Cross-continents		1 (1.0)	
**Country of the study**				
	United States		54 (51.9)	
	China		7 (6.7)	
	United Kingdom		6 (5.8)	
	Korea		6 (5.8)	
	Italy		0 (0)	
	Canada		2 (1.9)	
	Japan		1 (1.0)	
	Singapore		1 (1.0)	
	Netherlands		1 (1.0)	
	Australia		0 (0)	
	Hong Kong		0 (0)	
	Multiple countries		0 (0)	
	Others		26 (25.0)	
**Study design**				
	Randomized controlled trial		76 (73.1)	
	Cohort study		13 (12.5)	
		Prospective cohort study	7 (6.7)	
		Retrospective cohort study	6 (5.8)	
	Quasiexperimental study		9 (8.7)	
	Cross-sectional study		1 (1.0)	
	Single-arm trial		5 (4.8)	
**Patient population size**				
	≤500		90 (86.5)	
	501-1000		5 (4.8)	
	1001-5000		7 (6.7)	
	5001-10,000		0 (0)	
	10,001-50,000		1 (1.0)	
	Not specified		1 (1.0)	
**Type 2 diabetes subgroups**				
	Adult patients with diabetes (across all age ranges)		95 (91.3)	
	Elderly (≥65 years old)		3 (2.9)	
	Not specified		6 (5.8)	
**Study setting (health care)**				
	Primary		48 (46.2)	
	Tertiary		31 (29.8)	
	Mixed		4 (3.8)	
	Not applicable or not specified		21 (20.2)	
**Data source**				
	Primary		97 (93.3)	
	Secondary		4 (3.8)	
	Mixed (primary and secondary)		2 (1.9)	
	Not specified		1 (1.0)	
**Type of health coach**				
	Nurse		37 (35.6)	
	Multidisciplinary team		19 (18.3)	
	Diabetes educator		12 (11.5)	
	Researcher		5 (4.8)	
	Care coordinator		4 (3.8)	
	Dietician		4 (3.8)	
	Nonmedically trained coach (eg, peer coach)		4 (3.8)	
	Pharmacist		4 (3.8)	
	Medical doctor		2 (1.9)	
	Physiotherapist		1 (1.0)	
	Not specified		12 (11.5)	
**Duration of health coaching**				
	<1 month		0 (0)	
	1-3 months		25 (24.0)	
	4-6 months		24 (23.1)	
	7-12 months		15 (14.4)	
	1-2 years		7 (6.7)	
	3-4 years		2 (1.9)	
	≥5 years		1 (1.0)	
	Not specified		30 (28.8)	
**Remote monitoring modality**				
	Phone consultation		45 (43.3)	
	Automated devices/wearable devices		18 (17.3)	
	Teleconsultation		17 (16.3)	
	Digital applications/websites		17 (16.3)	
	Self-reported questionnaires		7 (6.7)	

### Clinical Outcomes

Table 2 shows the results from studies that evaluated clinical outcomes in patients with T2DM on remote monitoring who received health coaching. The clinical outcomes evaluated were divided into diabetes control, blood pressure control, blood lipid control, and other outcomes such as change in BMI or renal function.

**Table 2:** Studies that reported clinical outcomes.

Study	Remote monitoring modality	Duration of health coaching	Frequency of health coaching	Type of health coach	Role of the health coach	Details of the results
Kumar et al [27], 2018	In-app coaching and logging	3 months	5 times a week	Certified diabetes educator	To deliver supplemental content, support, encouragement, and accountability, and provide individualized feedback and insights based on logged data.	Significant improvement in HbA_1c_ (*P*<.001).
Wu et al [28], 2018	Telephone calls and automated voice system	12 months	1 time every month or every 3 months	Multidisciplinary team (NCMs^a^, nurse practitioners, physician, and social worker)	Supported care model involved in-person visits followed by telephone follow-ups. Technology-facilitated care model involved automated voice systems that were individually tailored for monitoring.	No significant improvement in HbA_1c_.
Grady et al [29], 2016	Telephone calls and web application	8 weeks	Once every 4 weeks	Not specified	To review data and provide management or lifestyle recommendations.	No significant improvement in HbA_1c_ (*P*>.05); some improvement of in-range BG^b^ (*P*=.001).
Hansen et al [30], 2017	Video teleconsultation and logging	8 months	Monthly	Nurse practitioner	To provide advice based on logged data.	Significant improvement in HbA_1c_ (*P*=.02), which became insignificant after 6 months; no significant improvement in lipid levels, BP^c^, BMI, and waist-hip ratio.
McFarland et al [31], 2012	Messaging device and logging	6 months	Ranges from daily to monthly	Clinical pharmacist specialist	To offer recommendations to increase lifestyle, compliance, and management of hypoglycemia, and change the insulin dosage.	Significant improvement in HbA_1c_ at 3 (*P*=.0002) and 6 months (*P*=.0066) but not in reduction from baseline to 6 months (*P*>.05).
Carter et al [32], 2011	Video teleconsultation and logging	Not specified	Biweekly	Therapist (nurse)	To discuss about self-management goals and behavior change strategies, and provide guidance on the data uploaded.	Significant reduction in HbA_1c_ (*P*<.05) and BMI (*P*<.05) but not BP (*P*>.05).
Toledo et al [33], 2014	Video teleconsultation and logging	6 months	3 times in 6 months	Endocrinologist and diabetes nurse educator	To update management and review self-monitoring BG data.	Significant reduction in HbA_1c_ (*P*<.05).
Emerson et al [34], 2015	Video teleconsultation and logging	3 months	Once or more weekly	Health coach	To provide ongoing diabetes education and reinforcement of the care plan, and facilitate virtual visits with providers on the multidisciplinary team.	Some reduction in HbA_1c_ (no *P* values).
Kim et al [35], 2005	Messaging	12 weeks	—^d^	Researcher (nursing college)	To provide optimal recommendations and continuous education, and ensure reinforcement of diet, exercise, medication, and monitoring.	Significant improvement in FPG^e^ (*P*=.006) and 2-h postprandial BG (*P*=.003); no significant improvement in lipid levels (*P*>.05).
Klobucar et al [36], 2012	Teleconsultation	—	—	Registered nurse	To monitor patients and provide diabetes education. Issues will be raised by a nurse to a physician and either will follow-up with the patient.	Significant improvement in HbA_1c_ (*P*<.001).
Brown et al [37], 2016	Telephone calls	—	—	Registered nurse	To provide real-time education, support, and coaching, and adjust medication according to the approved protocol.	Significant improvement in HbA_1c_ (*P*<.001).
Jordan et al [38], 2011	Telephone calls	—	1 to 2 times per month	Nurse	To give personalized care plans and updates.	Significant improvements in HbA_1c_, SBP^f^, DBP^g^, and BMI, but modest improvement in cholesterol levels, especially in patients with poor baseline values.
Jha et al [39], 2016	Telephone calls	—	Weekly	Diabetes educator	To assess glycemic control and troubleshoot any issues.	Significant improvements in HbA_1c_ (*P*=.001), fasting blood sugar (*P*=.000), and 2-h postprandial glucose (*P*=.000).
Tan et al [40], 2022	Telephone calls and logging	12 weeks	7 times over 12 weeks	Nurse and endocrinologist	To follow-up on FPG, insulin dose, medication management, lifestyle modification, and unsettled problems from past follow-ups.	Significant improvement in FPG (*P*<.001), especially in younger age groups.
Magee et al [41], 2021	Telephone calls, texts, emails, and logging	12 weeks	6 sessions of 1-2 weeks	Social worker	To provide more intense and frequent contact with participants, using real-time BG monitoring, remote visit offers, and T2DM^h^ medication management.	Significant improvement in HbA_1c_ (*P*<.001).
Areevut et al [42], 2022	Telephone calls	6 months	Every 3 months	Multidisciplinary team (advanced practice nurse, nurse, pharmacist, and dietician)	To provide assessment and education according to the framework of ADCES7 self-care behaviors.	Significant improvement in HbA_1c_ (*P*<.001) in both telehealth and in-person diabetes education, but no significant difference between them.
Nyenwe et al [43], 2022	Videoconference lessons and SMBG^i^	12 months	Every 3 months	Certified diabetes educator	To teach about the basic pathogenesis of diabetes, nutrition, physical activity, SMBG, effects of diabetes medications, sick day management, and complications of diabetes.	Significant improvement in HbA_1c_ (*P*=.009), but no significant difference compared to regular diabetes consultation; no significant improvements in BP and lipid levels.
Momin et al [44], 2022	Teleconsultation	3 months	Monthly	Nurse practitioner	To guide patients based on diabetes lifestyle interventions and assess the patient’s progress each month using the Plan-Do-Study-Act cycle.	Significant improvements in HbA_1c_ (*P*<.001) and eGFR^j^ (*P*<.001).
Nagrebetsky et al [45], 2013	Telephone calls and logging	12 months	Monthly	Research nursing staff	To ensure patient safety and help in titration, and provide supportive lifestyle interventions consisting of physical activity, diet, and medication changes.	Slightly greater improvement in HbA_1c_ with remote monitoring vs no remote monitoring (no *P* values provided).
Saslow et al [46], 2017	Videos	16 weeks	Weekly for 4 months and then biweekly for 4 months	—	To educate on adherence to a low-carb ketogenic diet and a behavioral adherence program.	Significant improvements in HbA_1c_ (*P*<.001), TG^k^ (*P*=.01), and body weight (*P*<.001); no significant improvements in HDL^l^ and LDL^m^ levels.
Dugas et al [47], 2018	Wearable device	13 weeks	—	Clinician	To view the patient’s behavior, trend, and score, and communicate with the patient.	Improvement in HbA_1c_ among individuals who had high adherence (*P*<.01).
Whitlock et al [48], 2000	Teleconsultation	3 months	Weekly and monthly	Case manager weekly; family physician monthly	To review goals, hypoglycemic episodes, and BG levels, and give recommendations for exercise, diet, nutrition, and well-being.	Significant improvements in HbA_1c_ (*P*<.05) and weight (*P*<.05) for the IG^n^ vs CG^o^.
Cohen et al [49], 2019	Telephone calls and logging	—	—	Pharmacist	To review telehealth data; educate on glucose, weight management, and positive reinforcement; and modify medications if needed.	No significant improvement in HbA_1c_ in pharmacist-led telehealth vs nurse-led telehealth.
de Vasconcelos et al [50], 2018	Telephone calls	6 months	—	Nurse	To provide guidance, motivate, encourage adherence to treatment, and follow-up on the proposed plan using therapeutic communication strategies.	No significant improvement in HbA_1c_ in remote coaching vs routine care; significant improvement in abdominal circumference in remote coaching (*P*=.001) vs routine care (*P*=.151); no significant improvements in BMI, waist-hip ratio, BP, fasting venous glucose, and lipid levels.
Kim et al [51], 2003	Telephone calls and logging	12 weeks	Twice a week for the 1st month and weekly for the 2nd and 3rd months	Nursing PhD student	To provide continued education, reinforcement of diet and exercise, and medication adjustment recommendations.	Significant improvement in HbA_1c_ levels in the IG vs CG.
Lee et al [52], 2015	Logging	5 weeks	Only if BG <3.9 mmol/L or >11.1 mmol/L	—	To provide feedback if BG levels fall outside of the range.	No significant improvements in FPG, serum fructosamine, and lipid levels.
Hsu et al [53], 2016	Teleconsultation and logging	12 weeks	—	Clinician coach	To coach on how to manage and titrate the insulin dose.	Significant improvement in HbA_1c_ in remote coaching vs standard care (*P*=.048).
Duruturk et al [54], 2019	Teleconsultation	6 weeks	Thrice a week	Physiotherapist	To provide telerehabilitation via breathing, calisthenic, rhythmic, strengthening, and stretching exercises.	Significant improvement in HbA_1c_ in telerehabilitation (*P*=.00) vs control (*P*=.23).
Crowley et al [55], 2016	Telephone calls and logging	6 months	Once every 2 weeks	Nurse	To review submitted SMBG data, reconcile medications, assess diabetes medication adherence, and deliver diabetes self-management support modules.	Significant improvement in HbA_1c_ in health coaching vs usual care (*P*=.05); significant improvements in SBP (*P*=.035) and DBP (*P*=.013).
Kim et al [56], 2007	Messaging and logging	6 months	—	Nurse	To provide personalized recommendations and encouragements based on uploaded data, and provide continuous education.	Significant improvements in HbA_1c_ and 2-h postmeal glucose in health coaching (*P*<.05) vs usual care; no significant improvement in FPG.
Kim et al [57], 2007	Messaging and logging	12 weeks	Weekly	Nurse	To provide personalized recommendations and encouragements based on uploaded data, and provide continuous education.	Significant improvements in HbA_1c_ and 2-h postmeal glucose in health coaching (*P*<.05) vs usual care; no significant improvement in FPG.
Kooiman et al [58], 2018	Messaging and logging	—	—	Diabetes nurse	To provide tailored feedback through SMS text messaging regarding activity, diet, exercise, and behavior, with the option to contact a nurse to ask questions.	No significant improvements in HbA_1c_, BMI, and waist-hip ratio; significant improvement in HbA_1c_ (*P*=.007) in responders vs nonresponders in the IG.
Ralston et al [59], 2009	Emails and logging	12 months	At least once a week	Care manager	To encourage participants to send BG readings weekly, respond to messages, review glucose levels, and adjust hypoglycemic medications.	Significant improvement in HbA_1c_ in health coaching vs usual care (*P*<.01); significant increase in the number of participants with HbA_1c_ <7% (*P*=.03).
Lee et al [60], 2017	Logging	12 weeks	—	Case manager	To provide advice on diabetes management, medication adherence, and lifestyle modification if BG levels fall outside of the range.	Significant improvements in HbA_1c_ and lipid control in telemonitoring vs usual care; no significant improvements in serum fructosamine, FPG, BP, and weight.
Greenwood et al [61], 2015	Telephone calls, in-app coaching, and logging	6 months	4th, 8th, and 12th weeks and daily health sessions	Certified diabetes educator	To discuss BG trends, goals, motivational interviewing, opportunities to improve, and brief educational content in health sessions.	No significant improvements in HbA_1c_ (*P*=.55) unless looking at instantaneous linear change (*P*=.005).
Kleinman et al [62], 2017	In-app coaching and logging	6 months	—	Health coach and provider	To regularly respond to patients’ questions (health coach), and to review the BG trend, lab results, and medications, and contact patients if needed (provider).	Significant improvement in HbA_1c_ for coaching vs usual care (*P*=.02); no significant improvements in FPG and BMI.
Sun et al [63], 2019	In-app coaching and logging	6 months	Medical team every 2 weeks; dietician monthly	Medical team and dietician	To send medical advice and reminders to patients and to provide dietary recommendations and guidance for BG monitoring based on the data logged.	Significant improvements in HbA_1c_ (*P*=.02) and postprandial glucose (*P*=.04) for coaching vs routine care; no significant improvements in lipid levels, BMI, and BP.
Zhou et al [64], 2014	In-app coaching and logging	—	—	Staff from the endocrine department	To provide guidance, key behavioral changes, and monitoring.	Significant improvements in HbA_1c_ (*P*<.001), fasting BG, BP, TG, and HDL in coaching vs usual care; no significant improvements in BMI, total cholesterol, and LDL.
Egede et al [65], 2017	Telephone calls and logging	—	If needed	Nurse	To follow-up on problematic patients or patients with abnormal results and adjust the algorithm under the supervision of the study physician and endocrinologist.	Significant improvements in HbA_1c_ (*P*=.024) and rate of decline in HbA_1c_ (*P*=.038) in coaching vs usual care.
von Storch et al [66], 2019	Telephone calls and logging	1 year	Monthly for the first 3 months, need-based thereafter	Coach (unspecified)	Staged program targeting diet, physical activity, self-control, emergency, clinical and stress management, and routine and mental training. To discuss and interpret data, and agree on goals for management.	Significant improvements in HbA_1c_ (*P*<.001) and BMI (*P*<.001).
Benson et al [67], 2019	Telephone calls	—	Monthly	PCP^p^ and RDN^q^	To carry out motivational interviewing, education, goal setting, and self-efficacy (PCP), and to provide medical nutrition therapy (RDN).	No significant improvements in HbA_1c_, BP, BMI, and LDL for coaching vs usual care.
Quinn et al [68], 2016	Messaging and logging	12 months	—	Virtual case manager	To send automated messages and personalized messages based on the data logged; self-care action plan every 2.5 months.	Evidence of improvement in HbA_1c_ with telehealth use in both younger and older patients (no *P* values provided).
Wang et al [69], 2019	In-app coaching	6 months	Weekly (first 3 months) and monthly (next 3 months) by a nurse; anytime by a physician	Diabetes specialist nurse and physician	To answer questions properly and promptly, as well as follow-up on personal health.	Significant improvements in HbA_1c_, FPG, and 2-h postprandial BG for coaching vs usual care (*P*<.05).
Clark et al [70], 2020	Messaging and logging	6 months	2 texts daily and calls if required	Study coordinator	To educate and monitor through motivation, education, or call to action.	Significant improvements in HbA_1c_ in medium/high distress groups (*P*<.001) but not in low/no distress groups (*P*=.065) for remote coaching vs usual care.
Stone et al [71], 2010	Telephone calls and logging	—	Monthly	Nurse practitioner	To provide further self-management education and counseling tailored to specific issues.	Significant improvements in HbA_1c_ levels in active care medication management vs care coordination (*P*<.001); no significant improvements in weight, BP, and lipid levels.
Wayne et al [72], 2015	In-app coaching and logging	6 months	At least once a week and on demand	Behavioral change counseling specialist	To guide healthy lifestyle choices and provide support when clients diverge from goals with the aim to reduce HbA_1c_, increase exercise, and modify diet.	Significant improvements in HbA_1c_ (*P*=.03) and BMI (*P*=.04) for telemonitoring vs no telemonitoring; no significant improvements in weight and waist circumference for telemonitoring vs no telemonitoring.
Yu et al [73], 2019	In-app coaching and logging	—	If needed	Clinician	To answer patients’ questions up to 8 h a day, 5 times a week with real-time communication, and to receive information of BG exceeding the safe range; virtual education program.	Significant improvements in patients with HbA_1c_ <7.0% in the 2 groups with in-app coaching vs usual care (*P*<.01); no significant improvements in FPG and 1,5-anhydroglucitol levels.
Quinn et al [74], 2011	In-app coaching and logging	—	Every 2.5 months	Diabetes educator	To provide automated management prompts, review data, and send supplemental messages (educators), and to provide a custom action plan every 2.5 months.	Significant improvements in HbA_1c_ in the coach-only (*P*=.003) and coach-PCP portal with decision support (*P*<.001) vs usual care; no significant improvements in BP and lipid levels.
Kempf et al [75], 2017	Telephone calls and logging	12 weeks	Weekly	Diabetes coach	To provide medication, healthy diet, PT, and lifestyle changes based on measured data via medical-mental motivation techniques and goal setting.	Significant improvements in HbA_1c_, body weight, BMI, BP, and 10-year cardiovascular disease risk (*P*<.05) for coaching vs usual care; no significant improvements in lipid levels.
Wang et al [76], 2017	In-app coaching and logging	6 months	Every 2 weeks	Medical team (not specified)	To review data and leave messages on site/phone call to supervise patients on self-monitoring, compliance, and exercise.	Significant improvements in HbA_1c_, FPG, postprandial BG, and TG (*P*<.05) for coaching vs usual care; no significant improvements in TC, HDL, LDL, BMI, and BP.
Gimbel et al [77], 2020	Messaging and logging	—	—	Research associate	To provide tailored behavioral messages to influence, activate, and reinforce behavior.	Significant improvements in HbA_1c_ (*P*=.006) and LDL (*P*=.01) for coaching vs usual care; no significant improvements in BMI, waist circumference, BP, and HDL.
Karhula et al [78], 2015	Telephone calls and logging	—	Every 4-6 weeks	Health coach	To evaluate mental and social conditions, health goals, and self-management, and discuss the results of SMBG.	No significant improvements in HbA_1c_, BP, lipid levels, and weight; significant improvements in waist circumference (*P*=.01).
Piette et al [79], 2001	Telephone calls and logging	12 months	Weekly	Nurse	To discuss symptoms, medication, and adherence, and follow-up on issues.	No significant improvements in HbA_1c_ and serum glucose.
Piette et al [80], 2000	Telephone calls and logging	1 year	1.4 times per month on average	Nurse	To address problems during assessment and provide general self-care education.	No significant improvement in HbA_1c_ (*P*=.1).
Heisler et al [81], 2019	Telephone calls	12 months	Minimum once a week	Peer coach	To provide educational content and facilitate goal setting and action plans.	Significant improvement in HbA_1c_ in both peer support–only and peer support + eHealth education groups, but no significant difference between the groups.
Anderson et al [82], 2010	Telephone calls	12 months	Varies between weekly, biweekly, and monthly depending on HbA_1c_ levels	Specialized nurse	To perform clinical assessment and discuss self-management (diet, exercise, stress, smoking, and goals), medication adherence, and glucose monitoring.	No significant improvements in HbA_1c_, BMI, BP, and LDL.
Nicolucci et al [83], 2015	Telephone calls and logging	12 months	Monthly	Nurse	To contact patients to discuss results/data and identify barriers to compliance or causes of inadequate control/pressure.	Significant improvement in HbA_1c_ (*P*=.001); no significant improvements in body weight, BP, and lipid levels.
Yasmin et al [84], 2020	Telephone calls	5 months	Every 10 days	—	To provide support on medication, diet, exercise, hospital visits, and other lifestyle modifications.	Significant improvements in fasting BG (*P*<.001) and 2-h postmeal BG (*P*<.001).
Bollyky et al [10], 2018	Messaging and logging	3 months	Daily	Certified diabetes educator	To provide algorithm-driven messages/encouragement with templated text message support. Involves personalized text messages, meal ratings, and activity recommendations for the intensive group.	Significant improvements in HbA_1c_ levels (*P*=.02), BG change (*P*=.02), and weight change (*P*=.02).
Jeong et al [85], 2018	Teleconsultation, logging, and messaging	24 weeks	Physician at 8 and 16 weeks; nurse when needed	Physician and diabetes specialist nurse	To provide automated short message feedback on glucose monitoring in the telemonitoring group and videoconferencing with physicians in the telemedicine group.	Significant improvement in HbA_1c_ in all groups, but no significant difference between IG and CG, except among those with >90% compliance; significant improvement in fasting BG for telemonitoring and telemedicine vs control; no significant improvements in lipid levels, body weight, and BP.
Forjuoh et al [86], 2014	Automated devices	6 weeks	—	Lay leader/experienced master trainer	To educate on diabetes self-management, decision making, and action planning.	Significant improvement in HbA_1c_ in all groups, but no significant difference between the IG and CG.
Tang et al [87], 2013	Messaging and logging	12 months	—	NCM and registered dietician	To check in and give feedback about data. To adjust medications based on the protocol and send educational messages (NCM).	Significant improvement in HbA_1c_ only at 6 months for the IG vs CG; significant improvement in LDL; no significant improvements in weight, BP, and Framingham risk.
Parsons et al [88], 2019	Telephone calls and logging	—	Monthly	Study nurse	To review BG readings and related events, and come up with goals and care plans.	Significant improvement in HbA_1c_ for the IG vs CG, but no significant improvement in HbA_1c_ change between telecare and SMBG only; significant improvement in waist circumference between telecare and control; no significant improvements in cholesterol, weight, and BMI across all groups.
Cho et al [89], 2017	Messaging, calls, and logging	6 months	Weekly for 3 months and alternate weeks for the next 3 months	Nurse	To provide additional individualized education for lifestyle management.	Significant improvements in HbA_1c_ (*P*<.001), postprandial glucose (*P*<.05), waist circumference reduction (*P*<.05), and body weight reduction (*P*<.01); no significant improvements in BMI, BP, fasting BG, and lipid levels.
Odnoletkova et al [90], 2016	Telephone calls	6 months	Every 5 weeks	Certified diabetes educator	To recommend and discuss lifestyle adjustments, adherence to therapy, knowledge, and training.	Significant improvements in HbA_1c_ (*P*=.001), total cholesterol (*P*=.001), BMI (*P*=.003), and weight (*P*=.004); at the 18-month follow-up, total cholesterol, BMI, and weight were no longer significant; no significant improvements in LDL, HDL, TG, and BP.
Lorig et al [91], 2010	In-app coaching and logging	6 months	Daily	Facilitator of peer support	To assist participants by reminding them to log on, modeling action planning and problem-solving, offering encouragement, and posting to bulletin boards.	Significant improvement in HbA_1c_ (*P*=.039) for the IG vs CG.
Izquierdo et al [92], 2010	Teleconsultation and logging	18 months	Monthly	Nurse and dietician	To determine patient interest to receive nutritional counseling from a dietician (nurse), and review data, facilitate behavioral change, facilitate medical nutrition therapy goals, etc (dietician).	No significant improvements in BMI (*P*=.063) and waist circumference (*P*=.602) in the IG vs CG; significant improvements in BMI (*P*=.004) and waist circumference (*P*=.006) when considering mediation effects of improved diet and exercise knowledge.
Shea et al [93], 2006	Telephone calls and logging	1 year	Every 4-6 weeks	NCM or dietician	To review BG and BP readings at each visit and assess progress. Discuss difficulties, and form a new goal.	Significant improvements in HbA_1c_ (*P*=.006), SBP (*P*=.001), DBP (*P*<.001), total cholesterol (*P*<.001), and LDL (*P*<.001) in the IG vs CG.
Shea et al [94], 2009	Telephone calls and logging	5 years	Every 4-6 weeks	NCM or dietician	To review BG and BP readings at each visit and assess progress. Discuss difficulties, and form a new goal.	Significant improvements in HbA_1c_ (*P*=.001), SBP (*P*=.024), DBP (*P*<.001), and lipid levels (*P*<.001) in the IG vs CG.
Crowley et al [95], 2022	Telephone calls and logging	12 months	Every 2 weeks	Nurse	To provide self-management education, review exercise progress, review medication management, and provide depression support.	Significant improvement in HbA_1c_ (*P*=.02) for comprehensive telehealth vs usual telemonitoring; no significant improvement in BMI.
Sayin Kasar et al [96], 2021	Telephone calls and messaging	12 weeks	Call every 2 weeks, message every week	Researcher	To provide education, send messages about general reminders, and make calls to discuss problems detected during the training; give information and answer questions.	Significant improvements in HbA_1c_ (*P*<.001), weight (*P*<.001), and SBP (*P*=.01) for the IG vs CG, but not in DBP.
Andreae et al [97], 2021	Telephone calls	6 months	Biweekly for 3 months, monthly for the next 3 months	Coach	To monitor progress, review educational content, and develop maintenance strategies for use after the program.	No significant improvements in HbA_1c_, SBP, LDL, and BMI.
Sherifali et al [98], 2019	Telephone calls	12 months	Weekly for 6 months, monthly for the next 6 months	Registered nurse and certified diabetes educator	To discuss topics, including care management and monitoring, self-management education, psychosocial support, and behavior modification.	Significant improvements in HbA_1c_ (*P*<.005), reduction of >0.5% in HbA_1c_ (*P*<.005), and HbA_1c_ <7% target (*P*<.005) for the IG vs CG.
St.-Jules et al [99], 2023	In-app coaching, videoconference lessons, and logging	6 months	Weekly for 4 weeks, every 2 weeks up to 20 weeks	Dietician	To provide personalized feedback reports on self-monitoring, diet, physical activity, and weight, and provide education- and behavior-focused PowerPoint presentations.	No significant improvements in HbA_1c_, BP, carotid-femoral pulse wave velocity, lipids, weight loss, urine Na:Cr, and urine P:Cr for the IG vs CG; significant improvements in weight loss in the first 3 months (*P*=.02) for monitoring vs nonmonitoring.
Esmaeilpour-BandBoni et al [100], 2021	Telephone calls	3 months	Weekly in the first month, every 2 weeks for the next 2 months	Nurse practitioner	To provide participants with education about 1 item from the diabetes educational package.	Significant improvement in HbA_1c_ (*P*<.001) for IG vs CG.
Sjattar et al [101], 2024	Telephone calls	2 months	8 weekly sessions	Nurse	To provide health education.	Significant improvement in fasting BG in the IG and CG, with no significant difference between the groups.
Tourkmani et al [102], 2024	Videoconference	3 months	Every 1-2 weeks	Certified diabetes educator and nurse	To discuss and review SMBG readings and guide toward proper insulin titrations and other injectable medications. To provide individualized diabetes education for hypo- or hyperglycemic events and encourage patients to ask any questions.	Significant improvement in HbA_1c_ (*P*<.001).
Ye at al [103], 2024	Text messages	26 weeks	Daily	Endocrinologist, cardiologist, and nurse	To provide daily posts on diabetes management, addressing questions, offering guidance on issues, and correcting unhealthy behaviors. Medical staff encouraged to share personal experiences to foster motivation and compliance.	Significant improvements in HbA_1c_, weight, BP, and cholesterol levels.
Mori et al [104], 2024	Videoconference	32 weeks	4 sessions over 32 weeks	Dietician	Four sessions on nutrition education conducted focusing on dietary management for glycemic control. Program was tailored to individual needs using the Nutrition Care Process Model, which includes assessment, diagnosis, intervention, and monitoring. Participants were also encouraged to set SMART goals for their dietary therapy.	Significant improvements in HbA_1c_ and weight in the IG and CG; noninferiority of telenutrition.
Berthoumieux et al [105], 2024	Web and mobile apps	2 years	Weekly; 104 lessons over 2 years	Health coach and certified diabetes care and education specialist (DCES)	To provide asynchronous support and allow for flexible communication and feedback (coach). The curriculum consists of 104 lessons delivered weekly over 2 years, covering diabetes management, lifestyle changes, and overall health topics. To encourage peer interaction through private forums.	Significant improvement in HbA_1c_ for baseline ≥8% vs baseline <8%, with both achieving significant improvements vs baseline; significant improvements in BMI and weight overall, with no significant difference between baseline ≥8% and <8%.
Josefsson et al [106], 2024	Mobile app	2 months	Weekly	Family physician	To provide feedback based on the submitted measurements weekly, and to check messages and data weekly and prompt participants who did not submit measurements.	No significant improvement in HbA_1c_.
Strombotne et al [107], 2023	Application	2 years	Onset and as needed based on participant progress	Certified nutritionist and dietitian	To provide guidance on a ketogenic diet, regular dietary advice, and medication management counseling and real-time logging of metrics, such as BG levels, for personalized adjustments.	Significant improvement in BMI; no significant improvements in HbA_1c_ and BP.
Gerber et al [108], 2024	Telehealth platform for messaging and phone calls	1 year of mHealth diabetes support followed by 1 year of usual care	Pharmacist encounter at least every 2-3 months; weekly monitoring by health coaches together with monthly home visits	Pharmacist and health coach trained specifically for the study	To provide remote support (clinical pharmacists) and health coach activities in person and through phone calls/text messaging. To compile home glucose monitoring data, share it with pharmacists, and provide ongoing support via text messaging (health coaches). To address barriers to medication use, assist with medication reconciliation, and provide diabetes self-management education (health coaches).	Significant improvement in HbA_1c_ (adjusted *P*=.005) that was sustained for a second year (adjusted *P*=.002); no significant improvements (adjusted) in BP, lipids, and BMI.
Tan et al [109], 2023	Video-based tele-education	6 months	Weekly educational sessions over 8 weeks and ongoing teleconsultation as required thereafter	Study nurse	To provide weekly reminders for self-monitoring and educational videos on T2DM management. Patients received immediate feedback from the app if their clinical parameters deviated beyond the stipulated range. Patients were prompted to verify their measurements and screen for related symptoms and medication adherence. To review their responses before taking appropriate actions, such as nurse-led teleconsultation or arranging for physician review on site (nurses).	Significant improvements in HbA_1c_, BP, and total cholesterol; no significant improvements in weight, BMI, other lipids, blood creatinine, eGFR, and urine albumin-creatinine ratio.
Jafar et al [110], 2023	Mobile phone app	3 months	Every 2 weeks and when necessary	Health coach (with a Master of Nursing degree)	To instruct participants to record their habits and measurements of BP and BG daily, and to make calls based on data input and emphasize the situations frequently observed when addressing behavior change in patients with T2DM. Patients determined health-related goals and monitored their progress with their coach.	Decreases in HbA_1c_ in both the IG and CG, but they were not significant.
Siminerio et al [111], 2023	Videoconference	12 months	Upon need and request	DCES	To provide diabetes self-management education and support where a comprehensive assessment is conducted, and to establish an individualized treatment plan with medication recommendations and self-management goals, with ongoing management and support by assessing progress, addressing challenges, and reinforcing self-care behaviors, problem solving skills, and coping strategies.	Significant improvements in HbA_1c_ in both the IG and CG, with no significant differences between the groups; significant association with a larger decrease in HbA_1c_ among those who met their self-management goals.
Kim et al [112], 2023	Automated devices	6 months	Every 2 weeks	Health coach	To discuss remote patient monitoring data, treatment goals and status, and adjustments to care plans every week. To reach out to enrolled patients every 2 weeks to engage in a patient-driven conversation about individual goals, self-management action plans, and any challenges faced.	Mean reduction in HbA_1c_ of 3.28 points at 3 months and 4.19 points at 6 months, with all remaining participants reaching or maintaining the target ≤8%; 10 of 15 remaining participants reached the systolic target of ≤130 and 8 reached the diastolic target of ≤80 at 6 months.
Dunkel et al [113], 2024	Phone calls	12 months	1 call per month	Diabetes coach	To provide devices for telemonitoring that automatically transfer data available to the diabetes coach, who supervises the patient and provides lifestyle interventions through individual and need-based telephone coaching (health specialists or diabetes coaches).	Significant improvement in HbA_1c_ for the IG vs CG at the end of the intervention and at the 24-month follow-up; significant improvement in BMI for the IG vs CG.
Gerber et al [108], 2024	Group discussions via online classrooms	22 weeks	Weekly: weeks 1-12, biweekly: weeks 13-16, monthly: weeks 17-22	Interventionist	The curriculum involved standardized, interactive materials to ensure consistency and engagement across group sessions. Each session followed a structured framework with personal sharing, peer advice, and goal-setting, supported by motivational interviewing techniques to encourage behavior change. Progress was reinforced in subsequent sessions to build commitment and cohesion, with visuals included to enhance relatability.	Significant weight changes in intervention participants after 12 and 17 sessions (*P*<.0001) and significant weight changes for the IG vs CG.
Apolzan et al [114], 2023	Phone app	24 weeks	Weekly	Not specified	To deliver the intervention weekly via virtual group workshops. Each workshop lasted 30-60 min and included a new topic related to building healthy habits, behavioral skills to support behavior change, and group discussion. Through the WW app and website, participants could track their weight, dietary intake, and physical activity; access progress reports; and complete weekly check-ins. The app also provided recipes, behavior change content, and T2DM-specific information.	Significant improvements in HbA_1c_, BMI, body weight, waist circumference, and DBP after 24 weeks; no significant improvement in SBP after 24 weeks.
Hoda et al [115], 2023	Text messages and telephone calls	3 months	Text message: 3-5 text messages weekly; telephone: once weekly	Trained pharmacist	The content of the text messages and calls aimed to enhance medication adherence, promote physical activity, encourage healthy eating habits, support smoking cessation, limit alcohol intake, and provide counseling on overall health. These text messages and telephone calls were provided by a trained pharmacist (research scholar) who ensured individualized delivery of the intervention.	Significant improvement in HbA_1c_ for the IG vs CG.
Rajkumar et al [116], 2023	Videoconference or telephone	Average of 159.3 days	Average of 5.7 times	Registered dietitian	Based on uploaded data, patients received feedback within 24 h regarding weight loss progress and any adjustments to be made to the nutritional or exercise plan and antidiabetic or antihypertensive medications.	Significant improvements in HbA_1c_, weight loss, BMI, SBP, and DBP, but no significant difference between the IG and CG.

^a^NCM: nurse care manager.

^b^BG: blood glucose.

^c^BP: blood pressure.

^d^Not applicable.

^e^FPG: fasting plasma glucose.

^f^SBP: systolic blood pressure.

^g^DBP: diastolic blood pressure.

^h^T2DM: type 2 diabetes mellitus.

^i^SMBG: self-monitoring blood glucose.

^j^eGFR: estimated glomerular filtration rate.

^k^TG: triglyceride.

^l^HDL: high-density lipoprotein.

^m^LDL: low-density lipoprotein.

^n^IG: intervention group.

^o^CG: control group.

^p^PCP: primary care physician.

^q^RDN: registered dietitian nutritionist.

#### Diabetes Control

Of the 92 studies that evaluated the impact of health coaching with remote monitoring on diabetes-related parameters, 68 (73%) showed improvements in diabetes-related parameters, such as HbA_1c_, fasting blood glucose, and postprandial blood glucose [10,27,30-43,45-48,51,53-56,59,60,62-77,81,83-91, 93-96,98,100,102-106,109,112,114,115]. In contrast, the remaining 19 studies showed no significant improvements in diabetes-related parameters [28,29,49,50,52,58,61,78-80,82, 97,99,101,107,108,110,111,116].

#### Blood Pressure Control

Pertaining to blood pressure control, majority of the studies (21/31, 68%) found no significant improvements in blood pressure [30,32,43,50,60,71,74,76,78,82,83,85,87,89,90,97, 99,103,107,108,116]. Only 10 studies found significant improvements in blood pressure [38,55,67,75,93,94, 96,109,112,114].

#### Blood Lipid Control

Among the 32 studies reporting outcomes on lipid-related parameters, 19 (59%) found no significant improvements in lipid levels [30,35,43,50-52,63,71,74,75,78,82,83,85,88,89, 97,99,109], 6 (19%) showed mixed evidence [46,50, 64,76,77,90], and 7 (22%) found significant improvements in lipid levels [46,60,67,87,93,94,103].

#### Other Clinical Outcomes

With regard to BMI changes, most studies (20/29, 69%) did not find any significant improvement in BMI [30,50,58,60,62-64,71,75,76,82,83,85,87-89,97,108,109,116].

Of the 2 studies that evaluated renal outcomes, 1 showed significant improvements in the estimated glomerular filtration rate (eGFR) [44] and 1 did not show any improvement [109]. Likewise, only 1 study evaluated mortality and showed a significant reduction in cardiovascular mortality [75].

### Humanistic Outcomes

Table 3 shows the results from studies that evaluated humanistic outcomes in patients with T2DM on remote monitoring who received health coaching. The humanistic outcomes evaluated were centered around quality of life, diabetes-related symptoms and distress, satisfaction with care, and other outcomes such as level of social support.

**Table 3:** Studies that reported humanistic outcomes.

Outcome and study author			Remote monitoring modality	Duration of health coaching		Frequency of health coaching		Type of health coach		Role of the health coach		Details of the result			
**Diabetes-related symptoms and distress**															
	Lee et al [52], 2015	Logging			5 weeks		Only if BG^a^ <3.9 mmol/L or >11.1 mmol/L		—^b^		To provide feedback if BG levels fall outside of the range.		Significant improvement in hypoglycemic risk during Ramadan (odds ratio 0.1273, 95% CI 0.0267-0.6059).		
	Kumar et al [27], 2018	In-app coaching and logging			3 months		5 times a week		Certified diabetes educator		To deliver supplemental content, support, encouragement, and accountability, and provide individualized feedback and insights based on logged data.		Significant improvement in Diabetes Distress Scale (DDS) scores (DDS-17) (*P*<.001), especially among those with high baseline DDS scores.		
	Jha et al [39], 2016	Telephone calls			—		Weekly		Diabetes educator		To assess glycemic control and troubleshoot any issues.		Lower documented episodes of hypoglycemia (15.3% for the IG^c^ vs 18.57% for the CG^d^).		
	Saslow et al [46], 2017	Videos			16 weeks		Weekly for 4 months and then biweekly for 4 months		—		To educate on adherence to a low-carb ketogenic diet and a behavioral adherence program.		No significant improvements in diabetes-related distress levels.		
	Lee et al [60], 2017	Logging			12 weeks		—		Case manager		To provide advice on diabetes management, medication adherence, and lifestyle modification if BG levels fall outside of the range.		Significant improvements in symptomatic hypoglycemia rates (*P*=.03); no significant improvements in diabetes distress assessments.		
	Kleinman et al [62], 2017	In-app coaching and logging			6 months		—		Health coach and provider		To regularly respond to patients’ questions (health coach), and to review the BG trend, lab results, and medications, and contact patients if needed (provider).		Significant improvement in diabetes distress/PAID-5 (*P*=.01).		
	Clark et al [70], 2020	Messaging and logging			6 months		2 texts daily and calls if required		Study coordinator		To educate and monitor through motivation, education, and call to action.		Significant improvement in the mean diabetes distress score (*P*<.001).		
	Quinn et al [74], 2011	In-app coaching and logging			—		Every 2.5 months		Diabetes educator		To provide automated management prompts, review data, and send supplemental messages (educators), and to provide a custom action plan every 2.5 months.		No significant improvements in the DDS score and diabetes symptom inventory.		
	Piette et al [79], 2001	Telephone calls and logging			12 months		Weekly		Nurse		To discuss symptoms, medication, and adherence, and follow-up on issues.		Significant improvement in overall diabetes-related symptoms (*P*=.04); no significant improvements in hyperglycemic, hypoglycemic, and vascular symptoms.		
	Piette et al [117], 2000	Telephone calls and logging			1 year		1.4 times per month on average		Nurse		To address problems during assessment and provide general self-care education.		Significant improvements in diabetes symptoms (*P*<.0001) and perceived glycemic control (*P*=.005).		
	Piette et al [80], 2000	Telephone calls and logging			12 months		Weekly		Nurse		To discuss symptoms, medication, and adherence, and follow-up on issues.		Significant improvement in the number of days in bed because of illness.		
	Jeong et al [85], 2018	Teleconsultation, logging, and messaging			24 weeks		Physician at the 8th and 16th weeks; nurse when needed		Physician and diabetes specialist nurse		To provide automated short message feedback on glucose monitoring in the telemonitoring group and videoconferencing with physicians in the telemedicine group.		Significant improvements in hypoglycemia-related events in the telemedicine group vs control group but not telemonitoring group.		
	Tang et al [87], 2013	Messaging and logging			12 months		—		NCM^e^ and registered dietician		To check in and give feedback about data. To adjust medications based on the protocol and send educational messages (NCM).		No significant differences in serious adverse events or adverse events such as hypoglycemia.		
	Crowley et al [95], 2022	Telephone calls and logging			12 months		Every 2 weeks		Nurse		To provide self-management education, review exercise progress, review medication management, and provide depression support.		Significant improvement in the DDS score (*P*<.007) for comprehensive telehealth vs usual telemonitoring.		
	Lorig et al [91], 2010	In-app coaching and logging			6 months		Daily		Facilitator of peer support		To assist participants by reminding them to log on, modeling action planning and problem-solving, offering encouragement, and posting to bulletin boards.		No significant improvements in health distress levels and activity limitation.		
	Tang et al [87], 2013	Messaging and logging			12 months		—		NCM and registered dietician		To check in and give feedback about data. To adjust medications based on the protocol and send educational messages (NCM).		No significant improvement in diabetes distress; significant improvement in the treatment distress score.		
	Trief et al [118], 2007	Telephone calls and logging			5 years		Every 4-6 weeks		NCM or dietician		To review BG and BP^f^ readings at each visit and assess progress. Discuss difficulties, and form a new goal.		No significant improvement in diabetes distress.		
	Li et al [119], 2018	Online forum			—		—		Multidisciplinary team		To engage in an interactive, theoretically-informed web-based self-management program for education.		Mean Problem Area in Diabetes (PAID) score was 1.9 (SE 1.3) lower for the IG vs CG.		
	Josefsson et al [106], 2024	Mobile app			2 months		Weekly		Family physician		To provide feedback based on the submitted measurements weekly, and to check messages and data weekly and prompt participants who did not submit measurements.		No significant improvement in the DDS score.		
	Gerber et al [108], 2024	Telehealth platform for messaging and phone calls			1 year of mHealth diabetes support followed by 1 year of usual care		Pharmacist encounter every 2-3 months; weekly monitoring and monthly home visits by coaches		Pharmacist and health coach trained specifically for the study		To provide remote support (clinical pharmacists) and health coach activities in person and through phone calls/text messaging. To compile home glucose monitoring data, share it with pharmacists, and provide ongoing support via text messaging (health coaches). To address barriers to medication use, assist with medication reconciliation, and provide diabetes self-management education (health coaches).		No significant improvements in diabetes distress levels.		
	Tan et al [109], 2023	Video-based tele-education			6 months		Weekly educational sessions over 8 weeks and ongoing teleconsultation as required thereafter		Study nurse		To provide weekly reminders for self-monitoring and educational videos on T2DM^g^ management. Patients received immediate feedback from the app if their clinical parameters deviated beyond the stipulated range. Patients were prompted to verify their measurements and screen for related symptoms and medication adherence. To review their responses before taking appropriate actions, such as nurse-led teleconsultation or arranging for physician review on site (nurses).		No significant improvements in PAID scores.		
	Siminerio et al [111], 2023	Videoconference			12 months		Upon need and request		Diabetes care and education specialist (DCES)		To provide diabetes self-management education and support where a comprehensive assessment was conducted, and to establish an individualized treatment plan with medication recommendations and self-management goals, with ongoing management and support by assessing progress, addressing challenges, and reinforcing self-care behaviors, problem solving skills, and coping strategies.		Significant improvements in regimen-related diabetes distress levels for those who met their self-management goals.		
	Apolzan et al [114], 2023	Phone app			24 weeks		Weekly		Not specified		To deliver the intervention weekly via virtual group workshops. Each workshop lasted 30-60 min and included a new topic related to building healthy habits, behavioral skills to support behavior change, and group discussion. Through the WW app and website, participants could track their weight, dietary intake, and physical activity; access progress reports; and complete weekly check-ins. The app also provided recipes, behavior change content, and T2DM-specific information.		Significant improvements in diabetes distress scores and Emotional Burden and Regimen Related Distress subscale scores.		
**Quality of life**															
	Wu et al [28], 2018	Telephone calls and automated voice system			12 months		Once every month or every 3 months		Multidisciplinary team (NCMs, nurse practitioners, physician, and social worker)		Supported care model involved in-person visits followed by telephone follow-ups. Technology-facilitated care model involved automated voice systems that were individually tailored for monitoring.		No significant improvements in exercise and general quality of life (SF-12) for technology-facilitated care vs the CG.		
	Hansen et al [30], 2017	Video teleconsultation and logging			8 months		Monthly		Nurse practitioner		To provide advice based on the logged data.		No significant improvement in general quality of life (SF-36).		
	Carter et al [32], 2011	Video teleconsultation and logging			Not specified		Biweekly		Therapist (nurse)		To discuss about self-management goals and behavior change strategies, and provide guidance on the data uploaded.		Significant improvement in perceived physical and mental health (*P*<.05).		
	Jha et al [39], 2016	Telephone calls			—		Weekly		Diabetes educator		To assess glycemic control and troubleshoot any issues.		Significant improvements in quality of life indices (*P*=.015).		
	Saslow et al [46], 2017	Videos			16 weeks		Weekly for 4 months and then biweekly for 4 months		—		To educate on adherence to a low-carb ketogenic diet and a behavioral adherence program.		No significant improvement in quality of life (SF-36).		
	Whitlock et al [48], 2000	Teleconsultation			3 months		Weekly and monthly		Case manager weekly; family physician monthly		To review goals, hypoglycemic episodes, and BG levels, and give recommendations for exercise, diet, nutrition, and well-being.		No significant improvements in diabetes quality of life and SF-36.		
	Lee et al [60], 2017	Logging			12 weeks		—		Case manager		To provide advice on diabetes management, medication adherence, and lifestyle modification if BG levels fall outside of the range.		No significant improvement in quality of life (EQ-5D).		
	Wayne et al [72], 2015	In-app coaching and logging			6 months		At least once a week and on demand		Behavioral change counseling specialist		To guide healthy lifestyle choices, provide support when the client diverges from goals with the aim to reduce HbA_1c_, increase exercise, and modify diet.		No significant improvements in satisfaction with life levels, the SF-12 physical score, and the SF-12 mental score.		
	Kempf et al [75], 2017	Telephone calls and logging			12 weeks		Weekly		Diabetes coach		To provide medication, healthy diet, PT, and lifestyle changes based on measured data via medical-mental motivation techniques and goal setting.		Significant improvements in quality of life (German Three-Factor-Eating-Questionnaire).		
	Karhula et al [78], 2015	Telephone calls and logging			—		Every 4-6 weeks		Health coach		To evaluate mental and social conditions, health goals, and self-management, and discuss the results of SMBG^h^.		No significant improvement in SF-36 health-related quality of life.		
	Piette et al [117], 2000	Telephone calls and logging			12 months		Weekly		Nurse		To discuss symptoms, medication, and adherence, and follow-up on issues.		No significant improvement in health-related quality of life.		
	Nicolucci et al [83], 2015	Telephone calls and logging			12 months		Monthly		Nurse		To contact patients to discuss results/data and identify barriers to compliance or causes of inadequate control/pressure.		Significant improvements in physical functioning, role emotional, mental health, and mental component summary scores; no significant improvements in other components of SF-36.		
	Li et al [119], 2018	Online forum			—		—		Multidisciplinary team		To engage in an interactive, theoretically-informed web-based self-management program for education.		Incremental quality-adjusted life years was 0.020 (95% CI –0.001 to 0.044) for the IG vs CG.		
	Andreae et al [97], 2021	Telephone calls			6 months		Biweekly for 3 months and monthly for the next 3 months		Coach		To monitor progress, review educational content, and develop maintenance strategies for use after the program.		No significant improvements in SF-12 mental and physical components.		
	Sherifali et al [98], 2019	Telephone calls			12 months		Weekly for 6 months and monthly for the next 6 months		Registered nurse and certified diabetes educator		To discuss topics, including care management and monitoring, self-management education, psychosocial support, and behavior modification.		Significant improvements in the 19-item Audit of Diabetes-Dependent Quality of Life (*P*<.005), diabetes QOL (*P*<.05), and present QOL (*P*<.05) mean change scores for coaching vs usual care.		
	Josefsson et al [106], 2024	Mobile app			2 months		Weekly		Family physician		To provide feedback based on the submitted measurements weekly, and to check messages and data weekly and prompt participants who did not submit measurements.		No significant improvements in EQ-5D-VAS scores.		
	Gerber et al [108], 2024	Telehealth platform for messaging and phone calls			1 year of mHealth diabetes support followed by 1 year of usual care		Pharmacist encounter every 2-3 months; weekly monitoring and monthly home visits by coaches		Pharmacist and health coach trained specifically for the study		To provide remote support (clinical pharmacists) and health coach activities in person and through phone calls/text messaging. To compile home glucose monitoring data, share it with pharmacists, and provide ongoing support via text messaging (health coaches). To address barriers to medication use, assist with medication reconciliation, and provide diabetes self-management education (health coaches).		No significant improvement in health-related quality of life.		
	Tan et al [40], 2022	Video-based tele-education			6 months		Weekly educational sessions over 8 weeks and ongoing teleconsultation as required thereafter		Study nurse		To provide weekly reminders for self-monitoring and educational videos on T2DM management. Patients received immediate feedback from the app if their clinical parameters deviated beyond the stipulated range. Patients were prompted to verify their measurements and screen for related symptoms and medication adherence. To review their responses before taking appropriate actions, such as nurse-led teleconsultation or arranging for physician review on site (nurses).		No significant improvements in EQ-5D-5L utility scores.		
	Jafar et al [110], 2023	Mobile phone app			3 months		Every 2 weeks and when necessary		Health coach (with a Master of Nursing degree)		To instruct participants to record their habits and measurements of BP and BG daily, and to make calls based on data input and emphasize the situations frequently observed when addressing behavior change in patients with T2DM. Patients determined health-related goals and monitored their progress with their coach.		Significant improvements in Indonesian Version of Diabetes Quality of Life-Brief Clinical Inventory (DQoL-BCI) scores for the CG vs IG.		
	Apolzan et al [114], 2023	Phone app			24 weeks		Weekly		Not specified		To deliver the intervention weekly via virtual group workshops. Each workshop lasted 30-60 min and included a new topic related to building healthy habits, behavioral skills to support behavior change, and group discussion. Through the WW app and website, participants could track their weight, dietary intake, and physical activity; access progress reports; and complete weekly check-ins. The app also provided recipes, behavior change content, and T2DM-specific information.		Significant improvements in the Impact of Weight on Quality of Life-Lite (IWQOL-L) overall score and all subscale scores.		
	Hoda et al [115], 2023	Text messages and telephone calls			3 months		Text message: 3-5 text messages weekly; telephone: once weekly		Trained pharmacist		The content of the text messages and calls aimed to enhance medication adherence, promote physical activity, encourage healthy eating habits, support smoking cessation, limit alcohol intake, and provide counseling on overall health. These text messages and telephone calls were provided by a trained pharmacist (research scholar) who ensured individualized delivery of the intervention.		Significant improvements in all components of the EQ-5D VAS, except for anxiety/depression in the IG.		
**Patient satisfaction**															
	Wu et al [28], 2018	Telephone calls and automated voice system			12 months		Once every month or every 3 months		Multidisciplinary team (NCMs, nurse practitioners, physician, and social worker)		Supported care model involved in-person visits followed by telephone follow-ups. Technology-facilitated care model involved automated voice systems that were individually tailored for monitoring.		Significant improvements in the levels of satisfaction with diabetes care and care for emotional problems (*P*=.05).		
	Kim et al [35], 2005	Messaging			12 weeks		—		Researcher (nursing college)		To provide optimal recommendations and continuous education, and ensure reinforcement of diet, exercise, medication, and monitoring.		Significant improvement in the patient care satisfaction score (*P*=.03).		
	Hsu et al [53], 2016	Teleconsultation and logging			12 weeks		—		Clinician coach		To coach on how to manage and titrate the insulin dose.		Significant improvement in the Diabetes Treatment Satisfaction Questionnaire for the IG vs CG (*P*=.01).		
	Kleinman et al [62], 2017	In-app coaching and logging			6 months		—		Health coach and provider		To regularly respond to patients’ questions (health coach), and to review the BG trend, lab results, and medications, and contact patients if needed (provider).		No significant improvement in general treatment satisfaction.		
	Piette et al [79], 2001	Telephone calls and logging			12 months		Weekly		Nurse		To discuss symptoms, medication, and adherence, and follow-up on issues.		Significant improvement in satisfaction with care.		
	Piette et al [117], 2000	Telephone calls and logging			12 months		Weekly		Nurse		To discuss symptoms, medication, and adherence, and follow-up on issues.		Significant improvement in satisfaction with care overall.		
	Cho et al [89], 2017	Messaging calls and logging			6 months		Weekly for 3 months and alternate weeks for the next 3 months		Nurse		To provide additional individualized education for lifestyle management.		Significant improvement in Diabetes Treatment Satisfaction Questionnaire status.		
	Tang et al [87], 2013	Messaging and logging			12 months		—		NCM and registered dietician		To check in and give feedback about data. To adjust medications based on the protocol and send educational messages (NCM).		Significant improvement in overall treatment satisfaction.		
	Siminerio et al [111], 2023	Videoconference			12 months		Upon need and request		DCES		To provide diabetes self-management education and support where a comprehensive assessment was conducted, and to establish an individualized treatment plan with medication recommendations and self-management goals, with ongoing management and support by assessing progress, addressing challenges, and reinforcing self-care behaviors, problem solving skills, and coping strategies.		High levels of acceptability with the intervention regardless of goal attainment based on the Telehealth Usability Questionnaire (TUQ).		
	Gerber et al [108], 2024	Group discussions via online classrooms			22 weeks		Weekly: weeks 1-12; biweekly: weeks 13-16; monthly: weeks 17-22		Interventionist		The curriculum involved standardized, interactive materials to ensure consistency and engagement across group sessions. Each session followed a structured framework with personal sharing, peer advice, and goal-setting, supported by motivational interviewing techniques to encourage behavior change. Progress was reinforced in subsequent sessions to build commitment and cohesion, with visuals included to enhance relatability.		High degree of enjoyment and acceptability of the program, indicating good program compatibility.		
	Bellido et al [120], 2023	Telephone			2 years		7 e-learning modules in 3 months followed by 11 touchpoints over the following 21 months		Nurse		The T-Coach program was a 2-year telemedicine educational tool to empower patients with T2DM treated with Gla-300 in terms of disease knowledge, self-management, and long-term adherence to treatment. The program consisted of e-learning modules and telephone sessions carried out by a team of nurses specialized in diabetes education.		Significant patient satisfaction with the T-Coach program.		
**Others**															
	Heisler et al [81], 2019	Telephone calls			12 months		Minimum once a week		Peer coach		To provide educational content and facilitate goal setting and action plans.		Significant improvements in diabetes-specific social support levels in both peer support only and peer support + eHealth education groups, but no significant differences between the groups.		
	Gerber et al [108], 2024	Telehealth platform for messaging and phone calls			1 year of mHealth diabetes support followed by 1 year of usual care		Pharmacist encounter every 2-3 months; weekly monitoring and monthly home visits by coaches		Pharmacist and health coach trained specifically for the study		To provide remote support (clinical pharmacists) and health coach activities in person and through phone calls/text messaging. To compile home glucose monitoring data, share it with pharmacists, and provide ongoing support via text messaging (health coaches). To address barriers to medication use, assist with medication reconciliation, and provide diabetes self-management education (health coaches).		No significant improvement in social support.		
	Motamed-Jahromi et al [121], 2024	Text and video messages			6 months		Daily for the first 2 months; weekly for subsequent months		Trained instructor		To provide educational content, including mindfulness training, according to self-care needs.		Significant improvements in diabetes self-regulation and elder self-neglect scale scores in the IG vs CG.		

^a^BG: blood glucose.

^b^Not applicable.

^c^IG: intervention group.

^d^CG: control group.

^e^NCM: nurse care manager.

^f^BP: blood pressure.

^g^T2DM: type 2 diabetes mellitus.

^h^SMBG: self-monitoring blood glucose.

#### Quality of Life

A total of 20 studies evaluated quality of life outcomes, where majority of the studies (12/20, 60%) found no improvement in quality of life outcomes [28,30,46,48,60,72,78,97,108, 109,117,119], 7 (35%) studies found significant improvements in patient quality of life [32,39,75,98,106,110,114,115], and 1 (5%) showed mixed evidence regarding the impact of health coaching with remote monitoring on patient quality of life [83].

#### Diabetes-Related Symptoms and Distress

A total of 21 studies reported on diabetes-related symptoms and distress experienced by patients. Approximately half of the studies (10/21, 48%) demonstrated improvement in diabetes-related symptoms and distress [27,39,52,62,80, 95,111,114,117,119]. Additionally, 4 (19%) studies showed mixed evidence for improvement [60,79,85,87], while 7 (33%) studies found no significant improvements in diabetes-related symptoms and distress [46,87,91,106,108,109,122].

#### Satisfaction With Care

A total of 11 studies reported on patient satisfaction with care, of which 10 (91%) studies found improvements in satisfaction with care [28,35,53,79,87,89,108,111,117,120], while only 1 (9%) study did not find significant improvements [62].

#### Other Humanistic Outcomes

Two studies explored the impact on the diabetes-related level of social support, and the evidence was mixed. One study showed significant improvements in social support levels [81], while another study showed no improvements [108].

### Psychiatric Outcomes

Table 4 shows the results from studies that evaluated psychiatric outcomes in patients with T2DM on remote monitoring who received health coaching. The psychiatric outcomes assessed across studies included depression and anxiety-related symptoms.

**Table 4:** Studies that reported psychiatric outcomes.

Study	Remote monitoring modality	Duration of health coaching	Frequency of health coaching	Type of health coach	Role of the health coach	Details of the results
Wu et al [28], 2018	Telephone calls and automated voice system	12 months	Once every month or every 3 months	Multidisciplinary team (NCMs^a^, nurse practitioners, physician, and social worker)	Supported care model involved in-person visits followed by telephone follow-ups. Technology-facilitated care model involved automated voice systems that were individually tailored for monitoring.	Significant improvements in depression symptoms (PHQ-9^b^) (*P*=.02), increased depression remission (*P*=.04), and Sheehan Disability Scale scores (*P*=.03) for technology-facilitated care vs the CG^c^.
Magee et al [41], 2021	Telephone calls, texts, email, and logging	12 weeks	6 sessions of 1- to 2-week intervals	Social worker	To provide more intense and frequent contact with participants, using real-time BG^d^ monitoring, remote visit offers, and T2DM^e^ medication management.	Significant improvements in depression levels/PHQ-9 (*P*=.01) and anxiety levels/GAD-7^f^ (*P*=.001).
Saslow et al [46], 2017	Videos	16 weeks	Weekly for 4 months and then biweekly for 4 months	—^g^	To educate on adherence to a low-carb ketogenic diet and a behavioral adherence program.	No significant improvements in CESD^h^ depression, CESD positive effect, DES^i^ negative effect, and DES positive effect.
Cohen et al [49], 2019	Telephone calls and logging	—	—	Pharmacist	To review telehealth data; educate on glucose, weight management, and positive reinforcement; and modify medications if needed.	No significant improvements in depression/CESD and anxiety/PHQ-9.
Duruturk et al [54], 2019	Teleconsultation	6 weeks	Thrice a week	Physiotherapist	To provide telerehabilitation via breathing, calisthenic, rhythmic, strengthening, and stretching exercises.	Significant improvement in the Beck depression scale in telerehabilitation vs control (*P*=.00).
Crowley et al [55], 2016	Telephone calls and logging	6 months	Once every 2 weeks	Nurse	To review submitted SMBG^j^ data, reconcile medications, assess diabetes medication adherence, and deliver diabetes self-management support modules.	No significant improvement in anxiety/PHQ-9.
Wayne et al [72], 2015	In-app coaching and logging	6 months	At least once a week and on demand	Behavioral change counseling specialist	To guide healthy lifestyle choices and provide support when clients diverge from goals with the aim to reduce HbA_1c_, increase exercise, and modify diet.	Significant improvement in mood/PANAS^k^ negative effect for telemonitoring vs no telemonitoring (*P*=.007); no significant improvements in HADS^l^ Anxiety, HADS Depression, and PANAS positive effect.
Quinn et al [74], 2011	In-app coaching and logging	—	Every 2.5 months	Diabetes educator	To provide automated management prompts, review data, and send supplemental messages (educators), and to provide a custom action plan every 2.5 months.	No significant improvement in depression/PHQ-9.
Piette et al [117], 2000	Telephone calls and logging	12 months	Weekly	Nurse	To discuss symptoms, medication, and adherence, and follow-up on issues.	Significant improvements in depression levels; no significant improvements in anxiety levels.
Kempf et al [75], 2017	Telephone calls and logging	12 weeks	Weekly	Diabetes coach	To provide medication, healthy diet, PT, and lifestyle changes based on measured data via medical-mental motivation techniques and goal setting.	Significant improvements in physical health (German CESD); no significant improvements in mental health (German CESD).
Lorig et al [91], 2010	In-app coaching and logging	6 months	Daily	Facilitator of peer support	To assist participants by reminding them to log on, modeling action planning and problem-solving, offering encouragement, and posting to bulletin boards.	No significant improvements in depression levels.
Crowley et al [95], 2022	Telephone calls and logging	12 months	Every 2 weeks	Nurse	To provide self-management education, review exercise progress, review medication management, and provide depression support.	No significant improvements in depression symptoms (*P*=.10) for comprehensive telehealth vs usual telemonitoring.
Tang et al [87], 2013	Messaging and logging	12 months	—	NCM and registered dietician	To check in and give feedback about data. To adjust medications based on the protocol and send educational messages (NCM).	No significant improvements in depression.
Trief et al [118], 2007	Telephone calls and logging	5 years	Every 4-6 weeks	NCM or dietician	To review BG and BP^m^ readings at each visit and assess progress. Discuss difficulties, and form a new goal.	No significant improvements in depression.
Gerber et al [108], 2024	Telehealth platform for messaging and phone calls	1 year of mHealth diabetes support followed by 1 year of usual care	Pharmacist encounter every 2-3 months; weekly monitoring and monthly home visits by coaches	Pharmacist and health coach trained specifically for the study	To provide remote support (clinical pharmacists) and health coach activities in person and through phone calls/text messaging. To compile home glucose monitoring data, share it with pharmacists, and provide ongoing support via text messaging (health coaches). To address barriers to medication use, assist with medication reconciliation, and provide diabetes self-management education (health coaches).	No significant improvements in depressive symptoms.

^a^NCM: nurse care manager.

^b^PHQ-9: Patient Health Questionnaire-9.

^c^CG: control group.

^d^BG: blood glucose.

^e^T2DM: type 2 diabetes mellitus.

^f^GAD-7: General Anxiety Disorder-7.

^g^Not applicable.

^h^CESD: Center for Epidemiologic Studies Depression Scale.

^i^DES: Differential Emotions Scale.

^j^SMBG: self-monitoring blood glucose.

^k^PANAS: Positive and Negative Affect Schedule.

^l^HADS: Hospital Anxiety and Depression Scale.

^m^BP: blood pressure.

#### Depression and Anxiety-Related Symptoms

A total of 15 studies assessed the psychiatric outcomes of patients. With regard to depression, approximately half of the studies (8/14, 58%) did not identify improvements in depression symptoms [49,72,74,87,91,95,108,118]. Only 5 (33%) studies showed significant improvements in depression symptoms [28,41,46,54,117], while 1 (7%) study showed mixed improvements in depression symptoms [75]. With regard to anxiety-related symptoms, majority of the studies (4/5, 80%) did not identify improvements in anxiety levels [49,55,72,117].

### Behavioral Outcomes

Table 5 shows the results from studies that evaluated behavioral outcomes in patients with T2DM on remote monitoring who received health coaching. The behavioral outcomes assessed comprised of diabetes self-efficacy and self-care, medication adherence, adherence to remote monitoring, and lifestyle modifications.

**Table 5:** Studies that reported behavioral outcomes.

Type of outcome presented and study			Remote monitoring modality		Duration of health coaching		Frequency of health coaching		Type of health coach		Role of the health coach		Details of the results		
**Diabetes self-care**															
	Wu et al [28], 2018	Telephone calls and automated voice system		12 months		Once every month or every 3 months		Multidisciplinary team (NCMs^a^, nurse practitioners, physician, and social worker)		Supported care model involved in-person visits followed by telephone follow-ups. Technology-facilitated care model involved automated voice systems that were individually tailored for monitoring.		No significant improvement in diabetes self-care.			
	Crowley et al [55], 2016	Telephone calls and logging		6 months		Once every 2 weeks		Nurse		To review submitted SMBG^b^ data, reconcile medications, assess diabetes medication adherence, and deliver diabetes self-management support modules.		Significant improvement in Self-Care Inventory Revised (SCI-R) (*P*=.047).			
	Greenwood et al [61], 2015	Telephone calls, in-app coaching, and logging		6 months		4th, 8th, and 12th weeks and daily health sessions		Certified diabetes educator		To discuss BG^c^ trends, goals, motivational interviewing, opportunities to improve, and brief educational content in health sessions.		Significant improvements in 3 out of 7 SDSCA^d^ subscales of foot care, carbohydrate spacing, and monitoring glucose.			
	Kleinman et al [62], 2017	In-app coaching and logging		6 months		—^e^		Health coach and provider		To regularly respond to patients’ questions (health coach), and to review the BG trend, lab results, and medications, and contact patients if needed (provider).		No significant improvements in diabetes self-care behaviors.			
	Wang et al [69], 2019	In-app coaching		6 months		Weekly for the first 3 months, monthly for the next 3 months by a nurse; anytime for a physician		Diabetes specialist nurse and physician		To answer questions properly and promptly, as well as follow-up on personal health.		Significant improvements in disease awareness and self-management ability (*P*<.05).			
	von Storch et al [66], 2019	Telephone calls and logging		1 year		Monthly for the first 3 months; need-based thereafter		Coach (unspecified)		Staged program targeting diet, physical activity, self-control, emergency, clinical and stress management, and routine and mental training. To discuss and interpret data, and agree on goals for management.		Significant improvements in the DSMQ^f^ (*P*=.000).			
	Crowley et al [95], 2022	Telephone calls and logging		12 months		Every 2 weeks		Nurse		To provide self-management education, review exercise progress, review medication management, and provide depression support.		Significant improvements in diabetes self-care (*P*<.001) for comprehensive telehealth vs usual telemonitoring.			
	Sayin Kasar et al [96], 2021	Telephone calls and messaging		12 weeks		Call every 2 weeks, message every week		Researcher		To provide education, send messages about general reminders, and make calls to discuss problems detected during the training; give information and answer questions.		Significant improvements in diabetes self-management (DSMQ) (*P*<.001) and perceived diabetes self-management (P-DSMQ) (*P*<.001).			
	Fernandes et al [123], 2016	Telephone calls		6 months		Average of 6 calls over 6 months		Nurse		To promote self-care, valorizing aspects, such as the autonomy of users regarding their choices, decision-making, and the development of a care plan.		Significant improvement in diabetes self-care questionnaire/ESM (*P*<.001).			
	Gimbel et al [77], 2020	Messaging and logging		—		—		Research associate		To provide tailored behavioral messages to influence, activate, and reinforce behavior.		No significant improvements in diabetes self-care activities.			
	Forjuoh et al [86], 2014	Automated devices		6 weeks		—		Lay leader/experienced master trainer		To educate on diabetes self-management, decision-making, and action planning.		No significant improvements in diabetes self-care activities.			
	Trief et al [122], 2013	Telephone calls and logging		5 years		Every 4-6 weeks		NCM or dietician		To review BG and BP^g^ readings at each visit and assess progress. Discuss difficulties, and form a new goal.		Significant improvement in self-care adherence for the IG^h^ vs CG^i^ (*P*<.001), but minority subjects were significantly consistently less adherent than white subjects; poorer adherence was predicted by greater comorbidity (*P*=.01) and diabetes symptoms (*P*<.001), while better adherence was predicted by greater duration of diabetes (*P*=.001) and more years of education (*P*=.002).			
	Piette et al [117], 2000	Telephone calls and logging		1 year		1.4 times per month on average		Nurse		To address problems during assessment and provide general self-care education.		Significant improvements in diabetes self-care, including more frequent glucose monitoring (*P*=.03), foot inspection (*P*=.02), and weight monitoring (*P*=.001).			
	Piette et al [79], 2001	Telephone calls and logging		12 months		Weekly		Nurse		To discuss symptoms, medication, and adherence, and follow-up on issues.		Significant improvements in home glucose monitoring (*P*=.05) and foot inspection (*P*=.05), but not in weight monitoring and medication problems.			
	Ye et al [103], 2024	Text messages		26 weeks		Daily		Endocrinologist, cardiologist, and nurse		To provide daily posts on diabetes management, addressing questions, offering guidance on issues, and correcting unhealthy behaviors. Medical staff encouraged to share personal experiences to foster motivation and compliance.		Significant improvements in total SDSCA scores and individual component scores.			
	Josefsson et al [106], 2024	Mobile app		2 months		Weekly		Family physician		To provide feedback based on the submitted measurements weekly, and to check messages and data weekly and prompt participants who did not submit measurements.		No significant improvements in DSMQ scores.			
	Gerber et al [108], 2024	Telehealth platform for messaging and phone calls		1 year of mHealth diabetes support followed by 1 year of usual care		Pharmacist encounter every 2-3 months; weekly monitoring and monthly home visits by coaches		Pharmacist and health coach trained specifically for the study		To provide remote support (clinical pharmacists) and health coach activities in person and through phone calls/text messaging. To compile home glucose monitoring data, share it with pharmacists, and provide ongoing support via text messaging (health coaches). To address barriers to medication use, assist with medication reconciliation, and provide diabetes self-management education (health coaches).		No significant improvements in diabetes self-care.			
	Tan et al [109], 2023	Video-based tele-education		6 months		Weekly educational sessions over 8 weeks and ongoing teleconsultation as required thereafter		Study nurse		To provide weekly reminders for self-monitoring and educational videos on T2DM^j^ management. Patients received immediate feedback from the app if their clinical parameters deviated beyond the stipulated range. Patients were prompted to verify their measurements and screen for related symptoms and medication adherence. To review their responses before taking appropriate actions, such as nurse-led teleconsultation or arranging for physician review on site (nurses).		Significant improvements in Self-Care Inventory-Revised (SCIR) scores.			
	Siminerio et al [111], 2023	Videoconference		12 months		Upon need and request		Diabetes care and education specialist (DCES)		To provide diabetes self-management education and support where a comprehensive assessment was conducted, and to establish an individualized treatment plan with medication recommendations and self-management goals, with ongoing management and support by assessing progress, addressing challenges, and reinforcing self-care behaviors, problem solving skills, and coping strategies.		Significant improvements in general diet diabetes self-care levels among those who met their self-management goals.			
**Diabetes self-efficacy**															
	Kumar et al [27], 2018	In-app coaching and logging		3 months		5 times a week		Certified diabetes educator		To deliver supplemental content, support, encouragement, and accountability, and provide individualized feedback and insights based on logged data.		Significant improvements in Diabetes Empowerment Scale (DES-SF) scores (*P*<.001).			
	Long et al [124], 2012	Telephone calls and logging		—		Varies between monthly, once every 7 weeks, and once every 3 months based on HbA_1c_		Telecarer (nonmedically trained)		To increase patient knowledge, understanding, self-management, and general self-care.		High levels of perceived empowerment in DES-SF (mean 4.25, 95% CI 4.17-4.33), and >90% expressed confidence in keeping their blood sugar controlled.			
	Zamanzadeh et al [125], 2017	Telephone calls and messaging		12 weeks		3 calls a week, daily messages		—		To conduct distance education and supportive interventions.		Significant improvements in overall self-empowerment, management of the psychosocial aspects of diabetes, dissatisfaction, and readiness to change, and setting and achieving diabetes goals for the IG vs CG (*P*<.001).			
	Lee et al [60], 2017	Logging		12 weeks		—		Case manager		To provide advice on diabetes management, medication adherence, and lifestyle modification if BG levels fall outside of the range.		No significant improvements in diabetes self-efficacy scale scores.			
	Greenwood et al [61], 2015	Telephone calls, in-app coaching, and logging		6 months		4th, 8th, and 12th weeks and daily health sessions		Certified diabetes educator		To discuss BG trends, goals, motivational interviewing, opportunities to improve, and brief educational content in health sessions.		No significant improvements in diabetes empowerment scale scores.			
	Kleinman et al [62], 2017	In-app coaching and logging		6 months		—		Health coach and provider		To regularly respond to patients’ questions (health coach), and to review the BG trend, lab results, and medications, and contact patients if needed (provider).		Significant improvement in diabetes self-efficacy (*P*=.04).			
	Sayin Kasar et al [96], 2021	Telephone calls and messaging		12 weeks		Calls every 2 weeks, message every week		Researcher		To provide education, send messages about general reminders, and make calls to discuss problems detected during the training; give information and answer questions.		Significant improvement in diabetes self-efficacy (*P*<.001) for the IG vs CG.			
	Bollyky et al [10], 2018	Messaging and logging		3 months		Daily		Certified diabetes educator		To provide algorithm-driven messages/encouragement with templated text message support. Involves personalized text messages, meal ratings, and activity recommendations for the intensive group.		No significant improvement in diabetes empowerment (DES-SF).			
	Andreae et al [97], 2021	Telephone calls		6 months		Biweekly for 3 months, monthly for the next 3 months		Coach		To monitor progress, review educational content, and develop maintenance strategies for use after the program.		Significant improvement in diabetes self-efficacy (*P*<.0001).			
	Piette et al [117], 2000	Telephone calls and logging		12 months		Weekly		Nurse		To discuss symptoms, medication, and adherence, and follow-up on issues.		Significant improvement in self-efficacy.			
	Lorig et al [91], 2010	In-app coaching and logging		6 months		Daily		Facilitator of peer support		To assist participants by reminding them to log on, modeling action planning and problem-solving, offering encouragement, and posting to bulletin boards.		Significant improvement in self-efficacy (*P*<.001) for the IG vs CG; significant improvement in PAM^k^ patient activation (*P*=.021) for the IG vs CG.			
	Crowley et al [95], 2022	Telephone calls and logging		12 months		Every 2 weeks		Nurse		To provide self-management education, review exercise progress, review medication management, and provide depression support.		Significant improvement in self-efficacy (*P*=.02) for comprehensive telehealth vs usual telemonitoring.			
	Gimbel et al [77], 2020	Messaging and logging		—		—		Research associate		To provide tailored behavioral messages to influence, activate, and reinforce behavior.		Significant improvement in patient activation measure scores (*P*=.007).			
	Anderson et al [82], 2010	Telephone calls		12 months		Varies between weekly, biweekly, and monthly depending on the HbA_1c_ level		Specialized nurse		To perform clinical assessment and discuss self-management (diet, exercise, stress, smoking, and goals), medication adherence, and glucose monitoring.		No significant improvements in perceived health status and physical assessment.			
	Gerber et al [108], 2024	Telehealth platform for messaging and phone calls		1 year of mHealth diabetes support followed by 1 year of usual care		Pharmacist encounter every 2-3 months; weekly monitoring and monthly home visits by coaches		Pharmacist and health coach trained specifically for the study		To provide remote support (clinical pharmacists) and health coach activities in person and through phone calls/text messaging. To compile home glucose monitoring data, share it with pharmacists, and provide ongoing support via text messaging (health coaches). To address barriers to medication use, assist with medication reconciliation, and provide diabetes self-management education (health coaches).		No significant improvement in diabetes self-efficacy.			
	Tan et al [109], 2023	Video-based tele-education		6 months		Weekly educational sessions over 8 weeks and ongoing teleconsultation as required thereafter		Study nurse		To provide weekly reminders for self-monitoring and educational videos on T2DM management. Patients received immediate feedback from the app if their clinical parameters deviated beyond the stipulated range. Patients were prompted to verify their measurements and screen for related symptoms and medication adherence. To review their responses before taking appropriate actions, such as nurse-led teleconsultation or arranging for physician review on site (nurses).		No significant improvement in PAM scores.			
	Siminerio et al [111], 2023	Videoconference		12 months		Upon need and request		DCES		To provide diabetes self-management education and support where a comprehensive assessment was conducted, and to establish an individualized treatment plan with medication recommendations and self-management goals, with ongoing management and support by assessing progress, addressing challenges, and reinforcing self-care behaviors, problem solving skills, and coping strategies.		No significant improvements in diabetes empowerment levels.			
**Medication adherence**															
	Cohen et al [49], 2019	Telephone calls and logging		—		—		Pharmacist		To review telehealth data; educate on glucose, weight management, and positive reinforcement; and modify medications if needed.		Significant improvement in cardiovascular, antidepressant, and overall medication adherence in pharmacist-led telehealth vs nurse-led telehealth; no significant improvement in diabetes medication/insulin and adjunct antidepressant adherence vs nurse-led telehealth.			
	Kim et al [51], 2003	Telephone calls and logging		12 weeks		Twice a week for the first month and weekly for the 2nd and 3rd months		Nursing PhD student		To provide continued education and ensure reinforcement of diet, exercise, medication, and adjustment recommendations.		No significant improvement in medication adherence.			
	Crowley et al [55], 2016	Telephone calls and logging		6 months		Once every 2 weeks		Nurse		To review submitted SMBG data, reconcile medications, assess diabetes medication adherence, and deliver diabetes self-management support modules.		No significant improvement in medication adherence.			
	Kleinman et al [62], 2017	In-app coaching and logging		6 months		—		Health coach and provider		To regularly respond to patients’ questions (health coach), and to review the BG trend, lab results, and medications, and contact patients if needed (provider).		Significant improvement in medication adherence (*P*=.03).			
	Benson et al [67], 2019	Telephone calls		—		Monthly		PCP^l^ and registered dietician nutritionist		To carry out motivational interviewing, education, goal setting, and self-efficacy (PCP), and to provide medical nutrition therapy (RDN^m^).		Significant improvement in diabetes medication adherence (*P*=.014), but not in cholesterol and BP medications.			
	Greenwood et al [61], 2015	Telephone calls, in-app coaching, and logging		6 months		4th, 8th, and 12th weeks and daily health sessions		Certified diabetes educator		To discuss BG trends, goals, motivational interviewing, opportunities to improve, and brief educational content in health sessions.		No significant improvement in the medication adherence component of the SDSCA measure.			
	Kempf et al [75], 2017	Telephone calls and logging		12 weeks		Weekly		Diabetes coach		To provide medication, healthy diet, PT, and lifestyle changes based on measured data via medical-mental motivation techniques and goal setting.		No significant improvement in antidiabetes medication adherence.			
	Piette et al [117], 2000	Telephone calls and logging		1 year		1.4 times per month on average		Nurse		To address problems during assessment and provide general self-care education.		Significant improvement in medication adherence (*P*=.003).			
	Yasmin et al [84], 2020	Telephone calls		5 months		Every 10 days		—		To provide support on medication, diet, exercise, hospital visits, and other lifestyle modifications.		No significant improvement in medication adherence.			
	Jeong et al [85], 2018	Teleconsultation, logging, and messaging		24 weeks		Physician at 8 and 16 weeks; nurse when needed		Physician and diabetes specialist nurse		To provide automated short message feedback on glucose monitoring in the telemonitoring group and videoconferencing with physicians in the telemedicine group.		Significant improvement in medication compliance for both telemonitoring and telemedicine groups vs control group.			
	Andreae et al [97], 2021	Telephone calls		6 months		Biweekly for 3 months, monthly for the next 3 months		Coach		To monitor progress, review educational content, and develop maintenance strategies for use after the program.		Significant improvement in medication adherence (*P*<.0001); significant improvement in medication use self-efficacy (*P*=.01).			
	Wungrath and Autorn [126], 2021	Telephone calls and videos		8 weeks		3 telephone calls and weekly videos		Researcher		To provide educational content about diabetes medication and conduct counseling sessions.		Significant improvements in medication adherence behavior (*P*<.001) and medication adherence knowledge (*P*<.001) for the IG vs CG.			
	Strombotne et al [107], 2024	Application		2 years		Onset and as needed based on participant progress		Certified nutritionist and dietitian		To provide guidance on a ketogenic diet, regular dietary advice, and medication management counseling and real-time logging of metrics, such as BG levels, for personalized adjustments.		Significant improvement in total monthly diabetes medication usage.			
	Gerber et al [108], 2024	Telehealth platform for messaging and phone calls		1 year of mHealth diabetes support followed by 1 year of usual care		Pharmacist encounter every 2-3 months; weekly monitoring and monthly home visits by coaches		Pharmacist and health coach trained specifically for the study		To provide remote support (clinical pharmacists) and health coach activities in person and through phone calls/text messaging. To compile home glucose monitoring data, share it with pharmacists, and provide ongoing support via text messaging (health coaches). To address barriers to medication use, assist with medication reconciliation, and provide diabetes self-management education (health coaches).		No significant improvement in medication adherence.			
	Tan et al [109], 2023	Video-based tele-education		6 months		Weekly educational sessions over 8 weeks and ongoing teleconsultation as required thereafter		Study nurse		To provide weekly reminders for self-monitoring and educational videos on T2DM management. Patients received immediate feedback from the app if their clinical parameters deviated beyond the stipulated range. Patients were prompted to verify their measurements and screen for related symptoms and medication adherence. To review their responses before taking appropriate actions, such as nurse-led teleconsultation or arranging for physician review on site (nurses).		Significant improvement in the “not taking the prescribed amount” section of the Medication Adherence Report Scale-5 (MARS-5).			
	Hoda et al [115], 2023	Text messages and telephone calls		3 months		Text message: 3-5 text messages weekly; telephone: once weekly		Trained pharmacist		The content of the text messages and calls aimed to enhance medication adherence, promote physical activity, encourage healthy eating habits, support smoking cessation, limit alcohol intake, and provide counseling on overall health. These text messages and telephonic calls were provided by a trained pharmacist (research scholar) who ensured individualized delivery of the intervention.		Significant improvement in medication compliance for the IG vs CG.			
	Bellido et al [120], 2023	Telephone		2 years		7 e-learning modules in 3 months followed by 11 touchpoints over the following 21 months		Nurse		The T-Coach program was a 2-year telemedicine educational tool to empower patients with T2DM treated with Gla-300 in terms of disease knowledge, self-management, and long-term adherence to treatment. The program consisted of e-learning modules and telephone sessions carried out by a team of nurses specialized in diabetes education.		No significant improvement in medication adherence.			
**Compliance with remote monitoring**															
	Dugas et al [47], 2018	Wearable device		13 weeks		—		Clinician		To view the patient’s behavior, trend, and score, and communicate with the patient.		No significant improvement in adherence to app usage.			
	Kim et al [51], 2003	Telephone calls and logging		12 weeks		Twice a week for the first month and weekly for the 2nd and 3rd months		Nursing PhD student		To provide continued education and ensure reinforcement of diet, exercise, medication, and adjustment recommendations.		Significant improvement in BG monitoring adherence; significant improvement in diet recommendation adherence; no significant improvements in exercise, foot care, and hypoglycemic management recommendation adherence.			
	Kooiman et al [58], 2018	Messaging and logging		—		—		Diabetes nurse		To provide tailored feedback through SMS text messaging regarding activity, diet, exercise, and behavior, with the option to contact a nurse to ask questions.		82.5% of intervention participants were determined to be adherent to the intervention program (they wore the Fitbit device on more than 75% of intervention days and read more than 50% of the program content).			
	Kleinman et al [62], 2017	In-app coaching and logging		6 months		—		Health coach and provider		To regularly respond to patients’ questions (health coach), and to review the BG trend, lab results, and medications, and contact patients if needed (provider).		Significant improvement in BG testing adherence (*P*=.01).			
	Bollyky et al [10], 2018	Messaging and logging		3 months		Daily		Certified diabetes educator		To provide algorithm-driven messages/encouragement with templated text message support. Involves personalized text messages, meal ratings, and activity recommendations for the intensive group.		No significant improvement in adherence to BG checks per day.			
	Parsons et al [88], 2019	Telephone calls and logging		—		Monthly		Study nurse		To review BG readings and related events, and come up with goals and care plans.		Adherence to SMBG (defined as having ≥80% of expected total SMBG readings) was seen in 71% of participants who completed the study.			
	Jeong et al [85], 2018	Teleconsultation, logging, and messaging		24 weeks		Physician at 8 and 16 weeks; nurse when needed		Physician and diabetes specialist nurse		To provide automated short message feedback on glucose monitoring in the telemonitoring group and videoconferencing with physicians in the telemedicine group.		No significant improvement in SMBG compliance.			
	Berthoumieux et al [105], 2024	Web and mobile app		2 years		Weekly; 104 lessons over 2 years		Health coach and certified DCES		To provide asynchronous support and allow for flexible communication and feedback (coach). The curriculum consists of 104 lessons delivered weekly over 2 years, covering diabetes management, lifestyle changes, and overall health topics. To encourage peer interaction through private forums.		50.8% (n=671) of members were classified as “highly engaged” with the program (median weekly actions ≥6; mean engagement=15.0 actions per week); program engagement remained high throughout most of the study in tandem with clinical improvement over 12 months.			
	Josefsson et al [106], 2024	Mobile app		2 months		Weekly		Family physician		To provide feedback based on the submitted measurements weekly, and to check messages and data weekly and prompt participants who did not submit measurements.		Significant improvement in adherence to BG tests per day.			
	Dunkel et al [113], 2024	Phone calls		12 months		1 call per month		Diabetes coach		To provide devices for telemonitoring that automatically transfer data available to the diabetes coach, who supervises the patient and provides lifestyle interventions through individual and need-based telephone coaching (health specialists or diabetes coaches).		High technology commitment and acceptance levels, with 60% of participants using devices or equivalents regularly after the intervention.			
	Gerber et al [108], 2024	Group discussions via online classrooms		22 weeks		Weekly: weeks 1-12; biweekly: weeks 13-16; monthly: weeks 17-22		Interventionist		The curriculum included standardized, interactive materials to ensure consistency and engagement across group sessions. Each session followed a structured framework with personal sharing, peer advice, and goal-setting, supported by motivational interviewing techniques to encourage behavior change. Progress was reinforced in subsequent sessions to build commitment and cohesion, with visuals included to enhance relatability.		High self-monitoring adherence: all intervention participants wore their fitness trackers daily, weighed themselves >3 times/week, and logged food >3 times/week.			
	Rajkumar et al [116], 2023	Videoconference or telephone		Average of 159.3 days		Average of 5.7 times		Registered dietitian		Based on uploaded data, patients received feedback within 24 h regarding weight loss progress and any adjustments to be made to the nutritional or exercise plan and antidiabetic or antihypertensive medications.		Significant reduction in dropout rates for the IG vs CG.			
**Diet**															
	Saslow et al [46], 2017	Videos		16 weeks		Weekly for 4 months and then biweekly for 4 months		—		To educate on adherence to a low-carb ketogenic diet and a behavioral adherence program.		Significant improvements in dietary carbs and sugar reduction (*P*<.001).			
	Benson et al [67], 2019	Telephone calls		—		Monthly		PCP and registered dietician nutritionist		To carry out motivational interviewing, education, goal setting, and self-efficacy (PCP), and to provide medical nutrition therapy (RDN).		Significant improvements in eating more fruits (*P*=.011) and wholegrains (*P*=.005), but not for vegetables.			
	Anderson et al [82], 2010	Telephone calls		12 months		Varies between weekly, biweekly, and monthly depending on the HbA_1c_ level		Specialized nurse		To perform clinical assessment and discuss self-management (diet, exercise, stress, smoking, and goals), medication adherence, and glucose monitoring.		No significant improvement in fruit/vegetable consumption.			
	Yasmin et al [84], 2020	Telephone calls		5 months		Every 10 days		—		To provide support on medication, diet, exercise, hospital visits, and other lifestyle modifications.		Significant improvements in carbohydrate, total calorie, vegetable, and fruit intake; no significant improvements in protein and fat intake.			
	Izquierdo et al [92], 2010	Teleconsultation and logging		18 months		Monthly		Nurse and dietician		To determine patient interest to receive nutritional counseling from a dietician (nurse), and review data, facilitate behavioral change, facilitate medical nutrition therapy goals, etc (dietician).		Significant improvement in diet (*P*=.002).			
	Kempf et al [75], 2017	Telephone calls and logging		12 weeks		Weekly		Diabetes coach		To provide medication, healthy diet, PT, and lifestyle changes based on measured data via medical-mental motivation techniques and goal setting.		Significant improvement in eating behavior (Framingham Risk Score).			
	Carter et al [32], 2011	Video teleconsultation and logging		Not specified		Biweekly		Therapist (nurse)		To discuss about self-management goals and behavior change strategies, and provide guidance on the data uploaded.		No improvement in the healthy eating scale score.			
	Mori et al [104], 2024	Videoconference		32 weeks		4 sessions over 32 weeks		Dietician		Four sessions on nutrition education conducted focusing on dietary management for glycemic control. Program was tailored to individual needs using the Nutrition Care Process Model, which includes assessment, diagnosis, intervention, and monitoring. Participants were also encouraged to set SMART goals for their dietary therapy.		Significant improvements in total energy, carbohydrate, and salt intake for both the IG and CG, showing noninferiority of telenutrition.			
	Hoda et al [115], 2023	Text messages and telephone calls		3 months		Text message: 3-5 text messages weekly; telephone: once weekly		Trained pharmacist		The content of the text messages and calls aimed to enhance medication adherence, promote physical activity, encourage healthy eating habits, support smoking cessation, limit alcohol intake, and provide counseling on overall health. These text messages and telephone calls were provided by a trained pharmacist (research scholar) who ensured individualized delivery of the intervention.		Significant improvement in eating habits for the IG vs CG.			
**Physical activity**															
	Kooiman et al [58], 2018	Messaging and logging		—		—		Diabetes nurse		To provide tailored feedback through SMS text messaging regarding activity, diet, exercise, and behavior, with the option to contact a nurse to ask questions.		Significant improvement in physical activity (*P*=.047).			
	von Storch et al [66], 2019	Telephone calls and logging		1 year		Monthly for the first 3 months, need-based thereafter		Coach (unspecified)		Staged program targeting diet, physical activity, self-control, emergency, clinical and stress management, and routine and mental training. To discuss and interpret data, and agree on goals for management.		No significant improvement in physical activity (*P*=.471).			
	Benson et al [67], 2019	Telephone calls		—		Monthly		PCP and registered dietician nutritionist		To carry out motivational interviewing, education, goal setting, and self-efficacy (PCP), and to provide medical nutrition therapy (RDN).		No significant improvement in getting more exercise.			
	Duruturk et al [54], 2019	Teleconsultation		6 weeks		Thrice a week		Physiotherapist		To provide telerehabilitation via breathing, calisthenic, rhythmic, strengthening, and stretching exercises.		Significant improvement in physical fitness in 5 out of 8 domains, and exercise capacity and muscle strength in 8 out of 10 domains.			
	Yasmin et al [84], 2020	Telephone calls		5 months		Every 10 days		—		To provide support on medication, diet, exercise, hospital visits, and other lifestyle modifications.		Significant improvement in adherence to exercise frequency per week, but not in mean hours per day.			
	Lorig et al [91], 2010	In-app coaching and logging		6 months		Daily		Facilitator of peer support		To assist participants by reminding them to log on, modeling action planning and problem-solving, offering encouragement, and posting to bulletin boards.		No significant improvement in aerobic exercise levels.			
	Izquierdo et al [92], 2010	Teleconsultation and logging		18 months		Monthly		Nurse and dietician		To determine patient interest to receive nutritional counseling from a dietician (nurse), and review data, facilitate behavioral change, facilitate medical nutrition therapy goals, etc (dietician).		Significant improvement in exercise (*P*=.002).			
	Carter et al [32], 2011	Video teleconsultation and logging		Not specified		Biweekly		Therapist (nurse)		To discuss about self-management goals and behavior change strategies, and provide guidance on the data uploaded.		No improvement in the physical activity scale score.			
	Josefsson et al [106], 2024	Mobile app		2 months		Weekly		Family physician		To provide feedback based on the submitted measurements weekly, and to check messages and data weekly and prompt participants who did not submit measurements.		No significant improvement in physical activity levels.			
	Tan et al [109], 2023	Video-based tele education		6 months		Weekly educational sessions over 8 weeks and ongoing teleconsultation as required thereafter		Study nurse		To provide weekly reminders for self-monitoring and educational videos on T2DM management. Patients received immediate feedback from the app if their clinical parameters deviated beyond the stipulated range. Patients were prompted to verify their measurements and screen for related symptoms and medication adherence. To review their responses before taking appropriate actions, such as nurse-led teleconsultation or arranging for physician review on site (nurses).		No significant improvement in the total physical activity metabolic equivalent of task (METS).			
	Dunkel et al [113], 2023	Phone calls		12 months		1 call per month		Diabetes coach		To provide devices for telemonitoring that automatically transfer data available to the diabetes coach, who supervises the patient and provides lifestyle interventions through individual and need-based telephone coaching (health specialists or diabetes coaches).		Significant improvement in physical activity in the IG vs CG.			
	Hoda et al [115], 2023	Text messages and telephone calls		3 months		Text message: 3-5 text messages weekly; telephone: once weekly		Trained pharmacist		The content of the text messages and calls aimed to enhance medication adherence, promote physical activity, encourage healthy eating habits, support smoking cessation, limit alcohol intake, and provide counseling on overall health. These text messages and telephonic calls were provided by a trained pharmacist (research scholar) who ensured individualized delivery of the intervention.		Significant improvement in physical activity in the IG.			
**Tobacco use**															
	Yasmin et al [84], 2020	Telephone calls		5 months		Every 10 days		—		To provide support on medication, diet, exercise, hospital visits, and other lifestyle modifications.		Significant improvement in adherence to tobacco control practice.			

^a^NCM: nurse care manager.

^b^SMBG: self-monitoring blood glucose.

^c^BG: blood glucose.

^d^SDSCA: Summary of Diabetes Self-Care Activities.

^e^Not applicable.

^f^DSMQ: Diabetes Self-Management Questionnaire.

^g^BP: blood pressure.

^h^IG: intervention group.

^i^CG: control group.

^j^T2DM: type 2 diabetes mellitus.

^k^PAM: patient activation measure.

^l^PCP: primary care physician.

^m^RDN: registered dietitian nutritionist.

#### Diabetes Self-Efficacy and Self-Care

A total of 19 studies explored the impact on diabetes self-efficacy, of which 12 (63%) reported significant improvements in diabetes self-efficacy [27,62,77,91,95-97, 103,111,117,124,125] and 7 (37%) reported no significant improvements [10,60,61,82,106,108,109]. Fourteen studies explored the impact on diabetes self-care adherence, of which 9 (64%) reported significant improvements in diabetes self-care adherence [55,66,69,80,95,96,109,122,123], 2 (14%) found mixed levels of improvement [61,79], and the remaining 6 (43%) found no significant improvements [28,62,77,86,108,111].

#### Medication Adherence

A total of 12 studies assessed self-reported adherence to diabetes medications. Evidence was mixed, with 9 (75%) studies reporting significant improvements [62,67,80,85,97,107,109, 115,126] and the remaining 8 (67%) reporting no significant improvements [49,51,55,61,75,84,108,120]. With regard to medication adherence for other chronic diseases, evidence was similarly mixed, with 1 study reporting improvement in medication adherence [49] and another study not reporting improvement [67].

#### Adherence to Remote Monitoring

A total of 12 studies reported on adherence to remote monitoring, such as measuring the blood glucose level. Of the 12 studies, 8 (67%) found that patients had a high rate of adherence to remote monitoring [58,62,88,105,106,108,113, 116], 1 (8%) reported mixed improvements [51], and the remaining 3 (25%) reported poor rates of adherence [10,47,85].

#### Lifestyle Modification

A total of 12 studies explored adherence to exercise. Majority of the studies (6/12, 50%) reported no significant improvement in exercise levels [32,66,67,91,106,109], 4 (33%) reported significant improvements in exercise levels [58,92,113,115], and 2 (17%) identified mixed improvements in exercise levels [54,84].

A total of 9 studies explored adherence to diet changes. Of the 9 studies, 5 (56%) reported improvements in diet [46,75,92,104,115], 2 (22%) found mixed improvements in diet [67,84], and the remaining 2 (22%) found no significant improvements in diet [32,82].

One study also reported significant improvements in the reduction of tobacco use [84].

### Knowledge Outcomes

Table 6 shows the results from studies that evaluated knowledge-related outcomes in patients with T2DM on remote monitoring who received health coaching. The knowledge-related outcomes evaluated primarily relate to diabetes-related knowledge.

**Table 6:** Studies that reported knowledge-related outcomes.

Study author	Remote monitoring modality	Duration of health coaching	Frequency of health coaching	Type of health coach	Role of the health coach	Details of the results
Carter et al [32], 2011	Video teleconsultation and logging	Not specified	Biweekly	Therapist (nurse)	To discuss about self-management goals and behavior change strategies, and provide guidance on the data uploaded.	Significant improvements in Diabetes Knowledge Scale scores (*P*<.05) and Diabetes Management Practices Scale scores (*P*<.05).
Jha et al [39], 2016	Telephone calls	—^a^	Weekly	Diabetes educator	To assess glycemic control and troubleshoot any issues.	Significant improvements in diabetes knowledge test scores (*P*=.005).
Lee et al [60], 2017	Logging	12 weeks	—	Case manager	To provide advice on diabetes management, medication adherence, and lifestyle modification if BG^b^ levels fall outside of the range.	No significant improvements in diabetes knowledge test scores.
Greenwood et al [61], 2015	Telephone calls, in-app coaching, and logging	6 months	4th, 8th, and 12th weeks and daily health sessions	Certified diabetes educator	To discuss BG trends, goals, motivational interviewing, opportunities to improve, and brief educational content in health sessions.	No significant improvements in diabetes knowledge test scores.
Kleinman et al [62], 2017	In-app coaching and logging	6 months	—	Health coach and provider	To regularly respond to patients’ questions (health coach), and to review the BG trend, lab results, and medications, and contact patients if needed (provider).	No significant improvements in diabetes knowledge scale scores.
Tang et al [87], 2013	Messaging and logging	12 months	—	NCM^c^ and registered dietician	To check in and give feedback about data. To adjust medications based on the protocol and send educational messages (NCM).	Significant improvements in knowledge about BG testing, knowledge about the disease, and willingness to recommend treatment.
Sjattar et al [101], 2024	Telephone calls	2 months	8 weekly sessions	Nurse	To provide health education.	Significant improvements in diabetes knowledge for the IG^d^ vs CG^e^.
Tan et al [109], 2023	Video-based tele-education	6 months	Weekly educational sessions over 8 weeks and ongoing teleconsultation as required thereafter	Study nurse	To provide weekly reminders for self-monitoring and educational videos on T2DM^f^ management. Patients received immediate feedback from the app if their clinical parameters deviated beyond the stipulated range. Patients were prompted to verify their measurements and screen for related symptoms and medication adherence. To review their responses before taking appropriate actions, such as nurse-led teleconsultation or arranging for physician review on site (nurses).	No significant improvements in Michigan Diabetes Knowledge Test (MDKT) scores.
Jafar et al [110], 2023	Mobile phone app	3 months	Every 2 weeks and when necessary	Health coach (with a Master of Nursing degree)	To instruct participants to record their habits and measurements of BP^g^ and BG daily, and to make calls based on data input and emphasize the situations frequently observed when addressing behavior change in patients with T2DM. Patients determined health-related goals and monitored their progress with their coach.	Significant improvements in Diabetes Knowledge Questionnaire-24 (DKQ-24) scores for the IG vs CG.
Bellido et al [120], 2023	Telephone	2 years	7 e-learning modules in 3 months followed by 11 touchpoints over the following 21 months	Nurse	The T-Coach program was a 2-year telemedicine educational tool to empower patients with T2DM treated with Gla-300 in terms of disease knowledge, self-management, and long-term adherence to treatment. The program consisted of e-learning modules and telephone sessions carried out by a team of nurses specialized in diabetes education.	Significant improvements in diabetes-related knowledge.

^a^Not applicable.

^b^BG: blood glucose.

^c^NCM: nurse care manager.

^d^IG: intervention group.

^e^CG: control group.

^f^T2DM: type 2 diabetes mellitus.

^g^BP: blood pressure.

#### Diabetes-Related Knowledge

A total of 9 studies explored the impact of health coaching with remote monitoring on diabetes-related knowledge. Of the 9 studies, 5 (56%) found significant improvements in diabetes-related knowledge [32,39,87,110,120] and the remaining 4 (44%) found no significant improvements [60-62,109].

### Economic Outcomes

Table 7 reports the results from studies that evaluated economic outcomes in patients with T2DM on remote monitoring who received health coaching. The economic outcomes assessed included health care–related costs and utilization.

**Table 7:** Studies that reported economic outcomes.

Outcome subcategory and study		Remote monitoring modality		Duration of health coaching	Frequency of health coaching	Type of health coach	Role of the health coach	Details of the results
**Health care utilization**								
	Hsu et al [53], 2016	Teleconsultation and logging	12 weeks		—^a^	Clinician coach	To coach on how to manage and titrate the insulin dose.	About 21% lower amount of time spent by the HCP^b^ per patient in the IG^c^ vs CG^d^.
	Ralston et al [59], 2009	Emails and logging	12 months		At least once a week	Care manager	To encourage participants to send BG^e^ readings weekly, respond to messages, review glucose levels, and adjust hypoglycemic medications.	No significant improvement in reduction of the number of outpatient visits, primary care provider visits, specialty physician visits, and inpatient days.
	Kleinman et al [62], 2017	In-app coaching and logging	6 months		—	Health coach and provider	To regularly respond to patients’ questions (health coach), and to review the BG trend, lab results, and medications, and contact patients if needed (provider).	No significant improvement in reduction of the frequency of communication with the doctor.
	Piette et al [79], 2001	Telephone calls and logging	12 months		Weekly	Nurse	To discuss symptoms, medication, and adherence, and follow-up on issues.	Significantly greater usage of specialty services (podiatry, diabetes clinic, cholesterol test, and medical foot exam) but not ophthalmology visits.
	Nicolucci et al [83], 2015	Telephone calls and logging	12 months		Monthly	Nurse	To contact patients to discuss results/data and identify barriers to compliance or causes of inadequate control/pressure.	No significant improvements in reduction of hospitalization rates, home visit rates, and specialist visit rates.
	Yasmin et al [84], 2020	Telephone calls	5 months		Every 10 days	—	To provide support on medication, diet, exercise, hospital visits, and other lifestyle modifications.	No significant improvement in hospital visits.
	Tang et al [87], 2013	Messaging and logging	12 months		—	NCM^f^ and registered dietician	To check in and give feedback about data. To adjust medications based on the protocol and send educational messages (NCM).	No significant improvement in total physician visits.
	Jia et al [127], 2009	Telephone calls	4 years		—	Nurse practitioner	To ask scripted questions about symptoms and health status.	Significant improvement in preventable hospitalization from long-term diabetes complications, lower limb amputations, and uncontrolled diabetes for the IG vs CG; no improvement in rehospitalization due to congestive heart failure, UTI^g^, COPD^h^, pneumonia, dehydration, angina, short-term diabetes complications, hypertension, and asthma.
	Wang et al [69], 2019	In-app coaching	6 months		Weekly for the first 3 months, monthly for the next 3 months by a nurse; anytime for a physician	Diabetes specialist nurse and physician	To answer questions properly and promptly, as well as follow-up on personal health.	Significant improvement in rehospitalization rates (*P*<.05).
	Strombotne et al [107], 2024	Application	2 years		Onset and as needed based on participant progress	Certified nutritionist and dietitian	To provide guidance on a ketogenic diet, regular dietary advice, and medication management counseling and real-time logging of metrics, such as BG levels, for personalized adjustments.	No significant improvements in outpatient visits, inpatient visits, or emergency department visits.
	Dunkel et al [113], 2024	Phone calls	12 months		1 call per month	Diabetes coach	To provide devices for telemonitoring that automatically transfer data available to the diabetes coach, who supervises the patient and provides lifestyle interventions through individual and need-based telephone coaching (health specialists or diabetes coaches).	The intervention had a temporary, group-specific effect on the number of physician contacts, with significant changes observed during and immediately after the intervention but a regression to baseline levels in the year following the intervention.
	Rajkumar et al [116], 2023	Videoconference or telephone	Average of 159.3 days		Average of 5.7 times	Registered dietitian	Based on uploaded data, patients received feedback within 24 h regarding weight loss progress and any adjustments to be made to the nutritional or exercise plan and antidiabetic or antihypertensive medications.	Significantly less appointments made per patient and decreased lost time and spent time in the IG vs CG.
**Cost**								
	Bollyky et al [10], 2018	Messaging and logging	3 months		Daily	Certified diabetes educator	To provide algorithm-driven messages/encouragement with templated text message support. Involves personalized text messages, meal ratings, and activity recommendations for the intensive group.	Estimated cost-saving attributable to HbA_1c_ reduction ($113-$179 per month) due to life coaching.
	Li et al [119], 2018	Online forum	—		—	Multidisciplinary team	To engage in an interactive, theoretically-informed web-based self-management program for education.	HeLP-Diabetes plus usual care was highly likely to be cost-effective. The incremental cost-effectiveness ratio was estimated at £5550 per QALY^i^ gained, with 87% and 92% probabilities of being cost-effective for willingness-to-pay thresholds of £20,000 and £30,000 per QALY, respectively.
	Mustonen et al [128], 2020	Telephone calls	12 months		10-11 times over 12 months	Coach (unspecified)	To have calls on disease awareness, medications, testing, control, lifestyle changes, risk control, strengths, and appointments.	Mean difference in the total cost per patient for patients with T2DM^j^ was 7% lower (–€3126) for the IG vs CG, but this was not statistically significant (*P*=.18); no significant improvements in the costs of secondary inpatient care and home care.
	Crowley et al [95], 2022	Telephone calls and logging	12 months		Every 2 weeks	Nurse	To provide self-management education, review exercise progress, review medication management, and provide depression support.	Approximately 2.6 times greater per-patient intervention cost for comprehensive telehealth vs usual telemonitoring.
	Strombotne et al [107], 2024	Application	2 years		Onset and as needed based on participant progress	Certified nutritionist and dietitian	To provide guidance on a ketogenic diet, regular dietary advice, and medication management counseling and real-time logging of metrics, such as BG levels, for personalized adjustments.	Significant associations with reductions in per-patient and per-month outpatient spending (–US $286.80 [SE 97.175]) and prescription drug costs (–US $105.40 [SE 30.332]).
	Dunkel et al [113], 2024	Phone calls	12 months		1 call per month	Diabetes coach	To provide devices for telemonitoring that automatically transfer data available to the diabetes coach, who supervises the patient and provides lifestyle interventions through individual and need-based telephone coaching (health specialists or diabetes coaches).	No significant main effect or significant interaction effect regarding cost.

^a^Not applicable.

^b^HCP: health care professional.

^c^IG: intervention group.

^d^CG: control group.

^e^BG: blood glucose.

^f^NCM: nurse care manager.

^g^UTI: urinary tract infection.

^h^COPD: chronic obstructive pulmonary disease.

^i^QALY: quality-adjusted life-year.

^j^T2DM: type 2 diabetes mellitus.

#### Health Care Costs

A total of 6 studies explored the impact of health coaching with remote monitoring on health care costs. Of the 6 studies, 3 (50%) showed that there were reductions in costs [10,107,119], 2 (33%) found no change in costs [113,128], and 1 (17%) reported increases in costs [95].

#### Health Care Utilization

A total of 11 studies reported on health care utilization rates. Majority of the studies (8/11, 73%) showed no significant difference in health care utilization, such as the number of specialty care provider visits and primary care visits [59,62,79,83,84,87,107,113].

### Summary of the Outcomes

**Figure 2:**
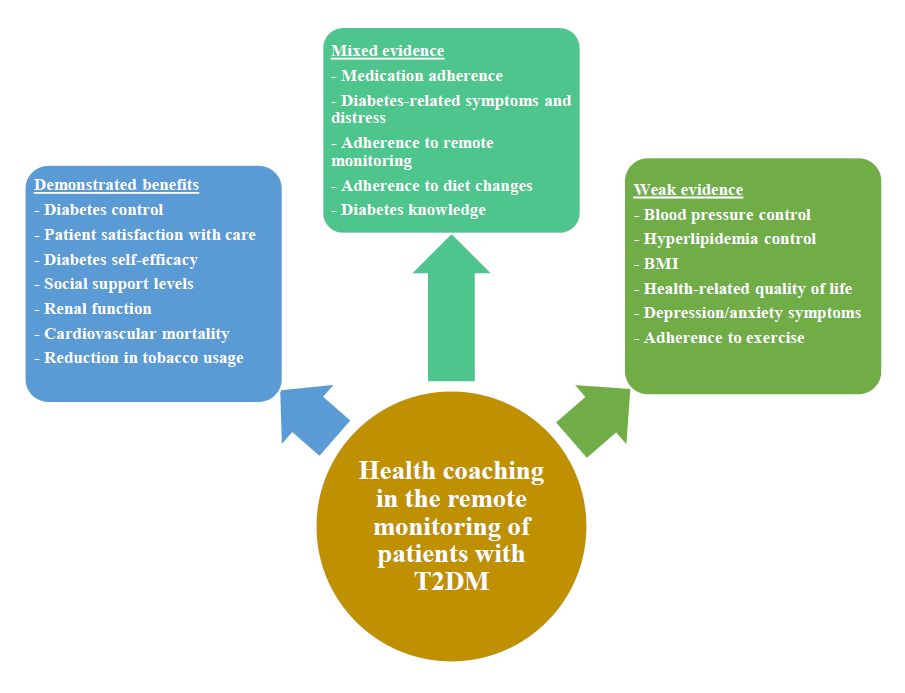
Summarized role of health coaching in the remote monitoring of patients with type 2 diabetes mellitus (T2DM).

Figure 2 depicts a simplified diagram summarizing the impact of health coaching on the remote monitoring of patients. Each outcome has been classified as “demonstrating benefits” (if majority of the studies showed improvement in the outcome), “mixed evidence” (if approximately equal number of studies showed benefits and lack of benefits), or “weak evidence” (if majority of the studies did not show improvement in the outcome).

## Discussion

### Summary and Discussion of Results

To the best of our knowledge, this is the first scoping review to evaluate the role of health coaching in the remote management of patients with T2DM. Overall, the current state of the literature suggests that health coaching is associated with improved outcomes, such as diabetes control and self-management skills, among patients with T2DM on remote monitoring.

The positive impact of health coaching on diabetes control and self-management in the remote monitoring of patients with T2DM is likely multifactorial. The management of T2DM requires a multipronged approach where effective self-management of diabetes, lifestyle modification, and medication adherence form the core tenets of care. High BMI, sedentary lifestyle, and unhealthy dietary patterns are well-established factors linked with poor T2DM control, which are amenable to lifestyle changes [129]. Through the establishment of “patient-centered, patient-driven” goals, digital health coaching serves as an important tool for the development and maintenance of sustainable and long-term behavioral change [130]. It also allows for the delivery of personalized health education via active learning techniques and provision of support to patients with psychosocial needs [131].

Interestingly, digital health coaching was not associated with improved control of blood lipid levels. This finding differed from that of another systematic review performed among patients with noncommunicable diseases, which showed that digital health coaching was associated with small to moderate effects in improving blood lipid levels [132]. There are few reasons that may explain the findings in this review. First, blood lipids constituted secondary outcomes in most of the included trials, and consequently, these studies may not be adequately powered to evaluate the effects of health coaching with remote monitoring on blood lipid levels. Additionally, individual study factors, such as baseline lipid levels, may play a role. For example, in the study by Andreae et al [97], patients in the intervention and study groups had generally well-controlled blood lipid levels (mean low-density lipoprotein cholesterol: 80.7 vs 84.6 mg/dL). In the context of relatively well-controlled blood lipids, the focus of health coaching in the remote monitoring of patients with T2DM in these studies may have diverted to target other chronic diseases requiring more attention. This in turn could have contributed to the muted effects of digital health coaching on blood lipid levels. Another postulated reason may be that existing remote monitoring technologies have inbuilt behavioral change prompts or cues, which may have attenuated the effects of health coaching [133].

With regard to blood pressure control, health coaching with remote monitoring was not associated with improved blood pressure control. This contrasted with findings from a review by Meng et al [134], which evaluated in-person health coaching among patients with hypertension and other chronic diseases. Our findings may be attributed to several factors. Of note, studies that evaluated blood pressure control after health coaching with remote monitoring were often underpowered to evaluate differences in blood pressure due to small sample sizes and attrition rates. Notably, the attrition rate was 33% in a study by Carter et al [32]. Additionally, blood pressure reduction from lifestyle modification is often seen only after 3 to 6 months, and the short follow-up period of studies included in this review may not have allowed for adequate assessment of the impact of health coaching via remote monitoring.

Mixed effects were noted with regard to the effects of health coaching on medication adherence among patients with T2DM on remote monitoring. This is unsurprising given the complex interplay of factors involved in medication adherence. Notably, the World Health Organization Framework has categorized factors affecting medication adherence into 5 main domains, namely, social and economic, health care team and system–related, condition-related, therapy-related, and patient-related dimensions [135]. While health coaching with remote monitoring may aid in targeting modifiable factors related to poor health literacy or behavior-related factors, it is less useful for patients with poor medication adherence secondary to physical limitations, such as cognitive impairment or poor dexterity [136]. Hence, careful patient selection is required to maximize the benefits from virtual health coaching. Some tools proposed for use in the literature, such as the Identification of Medication Adherence Barriers Questionnaire (IMAB-Q), may aid clinicians in appropriate patient identification [137].

Regarding psychiatric outcomes, health coaching via remote monitoring was not associated with improved depression or improved quality of life. This differed from the results of another review performed using in-person health coaching for chronic diseases [138]. Potential reasons may include the level of mental health training received by health coaching practitioners, as targeted coaching for psychiatric diseases usually requires different skillsets. In addition, mental wellness may not have been a priority for health coaches within the included studies, as the programs were primarily designed to target T2DM and its disease outcomes. Although studies have shown comparable outcomes between in-person and virtual health coaching [139], in-person health coaching may facilitate ease of expression of feelings and allow health coaches to use nonverbal cues to assess patients. Moving forward, these are potential limitations of using health coaching for patients with T2DM on remote monitoring. A combination of in-person and virtual health coaches to target specific subgroups of patients with T2DM, such as those with a high psychiatric disease burden, will help to achieve better outcomes.

With the increasing complexity of patients with T2DM having differing health care needs and uses [140], the involvement of other important stakeholders in the telemonitoring of patients with T2DM may aid in further optimizing patient outcomes. In a recent systematic review, the involvement of caregivers in the remote monitoring of patients with T2DM was shown to be associated with improvements in diabetes control and medication adherence [141]. Family caregivers have been shown to be beneficial in facilitating patients’ use of health technologies, especially among those who are less health literate, and improving their adherence to appointments [142]. This may have potential synergistic effects with health coaching involving remote monitoring and warrants further evaluation.

Despite the potential advantages of digital health coaching, challenges exist in the ecosystem currently, and health care administrators should be mindful of these challenges. There is currently a lack of evidence-based guidelines on the implementation of health coaching via remote monitoring platforms, especially with regard to the frequency and interval of health coaching [143]. Additionally, its implementation may possibly widen the digital divide for low-income communities or patients with poor digital health literacy due to their difficulties in acquiring or accessing technology devices to use digital health coaching services [144]. With the increasing wealth of literature on the topic, international medical societies should consider developing a set of guidelines and recommendations for the use of health coaching in the remote monitoring of patients and measures to deliver equitable care for less-privileged communities.

### Limitations

This scoping review has some limitations. First, due to significant heterogeneity in health coaching with remote monitoring and outcomes assessed across studies, meta-analyses were not performed, which should be considered in future reviews. The results from this scoping review will serve as potential precursors for more targeted systematic reviews and meta-analyses in the future to elucidate the benefits of health coaching and its role in the remote monitoring of patients with T2DM. Second, the outcomes assessed across studies lack standardization and limit comparisons between different studies. It is thus important for researchers to consider developing a core set of disease-specific outcomes that should be assessed in future studies evaluating the role of health coaching in the remote monitoring of patients with T2DM. A possible instrument that can be adapted is the CONSORT eHealth checklist, which has been used in general populations [145]. Third, risk of bias analyses were not performed as this scoping review was intended to provide an overview of the existing literature [146]. Nonetheless, we believe that the findings from this scoping review will guide researchers to develop more targeted systematic reviews to evaluate the benefits of health coaching in the remote monitoring of patients with T2DM, where a formal assessment of the risk of bias will be needed to obtain more critically appraised results. Lastly, the follow-up periods of most studies included in this review were short and spanned 1 year or less. The long-term outcomes from health coaching in the remote monitoring of patients with T2DM currently remain unclear, and studies with longer follow-up periods are required to provide insights into these outcomes.

### Conclusion

This scoping review has shown that health coaching plays a significant role in improving diabetes control and disease self-management among patients with T2DM on remote monitoring. Despite these promising results, evidence supporting its role in improving depression symptoms and medication adherence appears to be mixed, while that supporting its role in improving health care use, the control of blood lipid levels, and patient quality of life is weak. The potential reasons for the findings may be limited by interstudy differences in the type of health coaching with remote monitoring and the limited follow-up duration. Consequently, there is a need for further studies to elicit the optimal duration and type of health coaching with remote monitoring to obtain better outcomes in patients with T2DM.

## Appendix

Multimedia Appendix 1 Full search strategy.

Multimedia Appendix 2 Search query.

Multimedia Appendix 3 Inclusion and exclusion criteria using the PICOS (patient, intervention, comparator, outcomes, and study) framework.

Multimedia Appendix 4 Details of the included studies.

Multimedia Appendix 5 PRISMA-ScR (Preferred Reporting Items for Systematic Reviews and Meta-Analyses extension for Scoping Reviews) checklist.

## Abbreviations

T2DM: type 2 diabetes mellitus

## Data Availability

All data generated or analyzed in this study are included in this manuscript and its supplementary information files.
