# Supplementary material for: Health Coaching and Its Impact in the Remote Management of Patients With Type 2 Diabetes Mellitus: Scoping Review of the Literature
Source: J Med Internet Res. 2025 Apr 9;27:e60703. doi: 10.2196/60703 (PMC12018868; doi:10.2196/60703)
Supplement: Multimedia Appendix 1 [file jmir_v27i1e60703_app1.docx]

**Multimedia Appendix 1.** Full search strategy.

**1) Medline®**

Search strategy: #1 AND #2 AND #3

| **S/No.** | **Key themes** | **Terms** |
| --- | --- | --- |
| 1 | Type 2 Diabetes Mellitus | ("diabetes mellitus"[MeSH Terms] OR ("diabetes"[All Fields] AND "mellitus"[All Fields]) OR "diabetes mellitus"[All Fields] OR ("diabetes mellitus, type 2"[MeSH Terms] OR "type 2 diabetes mellitus"[All Fields] OR "diabetes mellitus type 2"[All Fields]) OR "T2DM"[All Fields] OR ("diabetes mellitus, type 2"[MeSH Terms] OR "type 2 diabetes mellitus"[All Fields]) OR ("diabetes mellitus, type 2"[MeSH Terms] OR "type 2 diabetes mellitus"[All Fields] OR "niddm"[All Fields] OR "niddms"[All Fields]) OR ("diabetes mellitus, type 2"[MeSH Terms] OR "type 2 diabetes mellitus"[All Fields] OR ("non"[All Fields] AND "insulin"[All Fields] AND "dependent"[All Fields] AND "diabetes"[All Fields] AND "mellitus"[All Fields]) OR "non insulin dependent diabetes mellitus"[All Fields])) |
| 2 | Health coaching | ((("health"[MeSH Terms] OR "health"[All Fields] OR "health s"[All Fields] OR "healthful"[All Fields] OR "healthfulness"[All Fields] OR "healths"[All Fields]) AND "coach*"[All Fields]) OR (("health"[MeSH Terms] OR "health"[All Fields] OR "health s"[All Fields] OR "healthful"[All Fields] OR "healthfulness"[All Fields] OR "healths"[All Fields]) AND ("belief s"[All Fields] OR "culture"[MeSH Terms] OR "culture"[All Fields] OR "belief"[All Fields] OR "beliefs"[All Fields])) OR ("health behaviour"[All Fields] OR "health behavior"[MeSH Terms] OR ("health"[All Fields] AND "behavior"[All Fields]) OR "health behavior"[All Fields]) OR (("health"[MeSH Terms] OR "health"[All Fields] OR "health s"[All Fields] OR "healthful"[All Fields] OR "healthfulness"[All Fields] OR "healths"[All Fields]) AND "manage*"[All Fields]) OR (("health"[MeSH Terms] OR "health"[All Fields] OR "health s"[All Fields] OR "healthful"[All Fields] OR "healthfulness"[All Fields] OR "healths"[All Fields]) AND "educat*"[All Fields]) OR (("health"[MeSH Terms] OR "health"[All Fields] OR "health s"[All Fields] OR "healthful"[All Fields] OR "healthfulness"[All Fields] OR "healths"[All Fields]) AND "promot*"[All Fields]) OR (("health"[MeSH Terms] OR "health"[All Fields] OR "health s"[All Fields] OR "healthful"[All Fields] OR "healthfulness"[All Fields] OR "healths"[All Fields]) AND "educat*"[All Fields]) OR (("health"[MeSH Terms] OR "health"[All Fields] OR "wellness"[All Fields]) AND "coach*"[All Fields]) OR (("life style"[MeSH Terms] OR ("life"[All Fields] AND "style"[All Fields]) OR "life style"[All Fields] OR "lifestyle"[All Fields] OR "lifestyles"[All Fields]) AND "coach*"[All Fields]) OR (("nurse s"[All Fields] OR "nurses"[MeSH Terms] OR "nurses"[All Fields] OR "nurse"[All Fields] OR "nurses s"[All Fields]) AND "manage*"[All Fields]) OR (("ieee int conf automation sci eng case"[Journal] OR "case phila"[Journal] OR "case"[All Fields]) AND "manage*"[All Fields]) OR (("prevent"[All Fields] OR "preventability"[All Fields] OR "preventable"[All Fields] OR "preventative"[All Fields] OR "preventatively"[All Fields] OR "preventatives"[All Fields] OR "prevented"[All Fields] OR "preventing"[All Fields] OR "prevention and control"[MeSH Subheading] OR ("prevention"[All Fields] AND "control"[All Fields]) OR "prevention and control"[All Fields] OR "prevention"[All Fields] OR "prevention s"[All Fields] OR "preventions"[All Fields] OR "preventive"[All Fields] OR "preventively"[All Fields] OR "preventives"[All Fields] OR "prevents"[All Fields]) AND "manage*"[All Fields]) OR (("prevent"[All Fields] OR "preventability"[All Fields] OR "preventable"[All Fields] OR "preventative"[All Fields] OR "preventatively"[All Fields] OR "preventatives"[All Fields] OR "prevented"[All Fields] OR "preventing"[All Fields] OR "prevention and control"[MeSH Subheading] OR ("prevention"[All Fields] AND "control"[All Fields]) OR "prevention and control"[All Fields] OR "prevention"[All Fields] OR "prevention s"[All Fields] OR "preventions"[All Fields] OR "preventive"[All Fields] OR "preventively"[All Fields] OR "preventives"[All Fields] OR "prevents"[All Fields]) AND "coach*"[All Fields]) OR (("motivate"[All Fields] OR "motivated"[All Fields] OR "motivates"[All Fields] OR "motivating"[All Fields] OR "motivation"[MeSH Terms] OR "motivation"[All Fields] OR "motivations"[All Fields] OR "motive"[All Fields] OR "motivational"[All Fields] OR "motivator"[All Fields] OR "motivators"[All Fields] OR "motives"[All Fields]) AND "interview*"[All Fields])) |
| 3 | Remote management | ("telemedicine"[MeSH Terms] OR "telemedicine"[All Fields] OR "telemedicine s"[All Fields] OR "tele-medicine"[All Fields] OR ("telehealth s"[All Fields] OR "telemedicine"[MeSH Terms] OR "telemedicine"[All Fields] OR "telehealth"[All Fields]) OR "tele-health"[All Fields] OR "telemonitor*"[All Fields] OR "tele monitor*"[All Fields] OR ("telemedicine"[MeSH Terms] OR "telemedicine"[All Fields] OR "ehealth"[All Fields]) OR (("electronical"[All Fields] OR "electronically"[All Fields] OR "electronics"[MeSH Terms] OR "electronics"[All Fields] OR "electronic"[All Fields]) AND ("health"[MeSH Terms] OR "health"[All Fields] OR "health s"[All Fields] OR "healthful"[All Fields] OR "healthfulness"[All Fields] OR "healths"[All Fields])) OR "e-health"[All Fields] OR ("telemedicine"[MeSH Terms] OR "telemedicine"[All Fields] OR ("mobile"[All Fields] AND "health"[All Fields]) OR "mobile health"[All Fields]) OR (("remote"[All Fields] OR "remotely"[All Fields] OR "remoteness"[All Fields] OR "remotes"[All Fields]) AND "manag*"[All Fields]) OR (("remote"[All Fields] OR "remotely"[All Fields] OR "remoteness"[All Fields] OR "remotes"[All Fields]) AND "monitor*"[All Fields]) OR (("remote"[All Fields] OR "remotely"[All Fields] OR "remoteness"[All Fields] OR "remotes"[All Fields]) AND "consult*"[All Fields]) OR ("mhealth s"[All Fields] OR "telemedicine"[MeSH Terms] OR "telemedicine"[All Fields] OR "mhealth"[All Fields]) OR "m-health"[All Fields] OR ("lancet digit health"[Journal] OR "digit health"[Journal] OR ("digital"[All Fields] AND "health"[All Fields]) OR "digital health"[All Fields]) OR ("telematic"[All Fields] OR "telematics"[All Fields]) OR "telemanag*"[All Fields] OR "tele manag*"[All Fields] OR "tele nurs*"[All Fields] OR "telenurs*"[All Fields] OR "teleservic*"[All Fields] OR "tele servic*"[All Fields] OR "tele-care"[All Fields] OR "telecare"[All Fields] OR "Tele-home"[All Fields] OR "telehome"[All Fields] OR "tele conferenc*"[All Fields] OR "teleconferenc*"[All Fields] OR "teleconsult*"[All Fields] OR "tele consult*"[All Fields] OR "telecommunicat*"[All Fields] OR "tele communicat*"[All Fields]) |

**2) Embase®**

Search strategy: #1 AND #2 AND #3

| **S/No.** | **Key themes** | **Terms** |
| --- | --- | --- |
| 1 | Type 2 Diabetes Mellitus | ('diabetes mellitus':ti,ab,kw OR 'diabetes mellitus, type 2':ti,ab,kw OR t2dm:ti,ab,kw OR 'type 2 diabetes mellitus':ti,ab,kw OR niddm:ti,ab,kw OR 'non-insulin dependent diabetes mellitus':ti,ab,kw) |
| 2 | Health coaching | ('health coach*':ti,ab,kw OR 'health belief':ti,ab,kw OR 'health behaviour':ti,ab,kw OR 'health manage*':ti,ab,kw OR 'health promot*':ti,ab,kw OR 'health educat*':ti,ab,kw OR 'wellness coach*':ti,ab,kw OR 'lifestyle coach*':ti,ab,kw OR 'nurse manage*':ti,ab,kw OR 'case manage*':ti,ab,kw OR 'prevention manage*':ti,ab,kw OR 'prevention coach*':ti,ab,kw OR 'motivational interview*':ti,ab,kw) |
| 3 | Remote management | (telemedicine:ti,ab,kw OR 'tele medicine':ti,ab,kw OR telehealth:ti,ab,kw OR 'tele health':ti,ab,kw OR telemonitor*:ti,ab,kw OR 'tele monitor*':ti,ab,kw OR ehealth:ti,ab,kw OR 'electronic health':ti,ab,kw OR 'e health':ti,ab,kw OR 'mobile health':ti,ab,kw OR 'remote manag*':ti,ab,kw OR 'remote monitor*':ti,ab,kw OR 'remote consult*':ti,ab,kw OR mhealth:ti,ab,kw OR 'm health':ti,ab,kw OR 'digital health':ti,ab,kw OR telematic:ti,ab,kw OR 'tele matic':ti,ab,kw OR telemanag*:ti,ab,kw OR 'tele manag*':ti,ab,kw OR 'tele nurs*':ti,ab,kw OR telenurs*:ti,ab,kw OR teleservic*:ti,ab,kw OR 'tele servic*':ti,ab,kw OR 'tele care':ti,ab,kw OR telecare:ti,ab,kw OR 'tele home':ti,ab,kw OR telehome:ti,ab,kw OR 'tele conferenc*':ti,ab,kw OR teleconferenc*:ti,ab,kw OR teleconsult*:ti,ab,kw OR 'tele consult*':ti,ab,kw OR telecommunicat*:ti,ab,kw OR 'tele communicat*':ti,ab,kw) |

**3) CINAHL®**

Search strategy: #1 AND #2 AND #3

| **S/No.** | **Key themes** | **Terms** |
| --- | --- | --- |
| 1 | Type 2 Diabetes Mellitus | ((Diabetes mellitus) OR (Diabetes mellitus, type 2) OR (T2DM) OR (Type 2 diabetes mellitus) OR (NIDDM) OR (non-insulin dependent diabetes mellitus)) |
| 2 | Health coaching | ((health coach*) OR (health belief) OR (health behaviour) OR (health manage*) OR (health educat*) OR (health promot*) OR (health educat*) OR (wellness coach*) OR (lifestyle coach*) OR (nurse manage*) OR (case manage*) OR (prevention manage*) OR (prevention coach*) OR (motivational interview*)) |
| 3 | Remote management | (((Telemedicine) OR (tele-medicine) OR (telehealth) OR (tele-health) OR (Telemonitor*) OR (tele-monitor*) OR (ehealth) OR (electronic health) OR (e-health) OR (mobile health) OR (remote manag*) OR (remote monitor*) OR (remote consult*) OR (mhealth) or (m-health) OR (digital health) OR (Telematic) OR (tele-matic) OR (telemanag*) OR (Tele-manag*) OR (tele-nurs*) OR (telenurs*) OR (teleservic*) OR (tele-servic*) OR (tele-care) OR (telecare) OR (Tele-home) OR (telehome) OR (Tele-conferenc*) OR (Teleconferenc*) OR (Teleconsult*) OR (Tele-consult*) OR (telecommunicat*) OR (tele-communicat*))) |

**4) PsycInfo®**

| **S/No.** | **Key themes** | **Terms** |
| --- | --- | --- |
| 1 | Type 2 Diabetes Mellitus | ((Diabetes mellitus) OR (Diabetes mellitus, type 2) OR (T2DM) OR (Type 2 diabetes mellitus) OR (NIDDM) OR (non-insulin dependent diabetes mellitus)) |
| 2 | Health coaching | ((health coach*) OR (health belief) OR (health behaviour) OR (health manage*) OR (health educat*) OR (health promotion) OR (health education) OR (wellness coach*) OR (lifestyle coach*) OR (nurse manage*) OR (case manage*) OR (prevention manage*) OR (prevention coach*) OR (motivational interview*)) |
| 3 | Remote management | ((Telemedicine) OR (telehealth) OR (Telemonitor) OR (tele-monitor) OR (Telemonitoring) OR (Tele-monitoring) OR (ehealth) OR (electronic health) OR (e-health) OR (mobile health) OR (remote management) OR (remote monitoring) OR (remote consultation) OR (mhealth) or (m-health) OR (digital health) OR (Telematic) OR (tele-matic) OR (telemanagement) OR (Tele-management) OR (tele-nursing) OR (telenursing) OR (teleservic*) OR (tele-servic*) OR (tele-care) OR (telecare) OR (Tele-home) OR (telehome) OR (Tele-conferenc*) OR (Teleconferenc*) OR (Teleconsult) OR (Tele-consult) OR (telecommunication) OR (tele-communication)) |

**5) Web of Science**

| **S/No.** | **Key themes** | **Terms** |
| --- | --- | --- |
| 1 | Type 2 Diabetes Mellitus | ((Diabetes mellitus) OR (Diabetes mellitus, type 2) OR (T2DM) OR (Type 2 diabetes mellitus) OR (NIDDM) OR (non-insulin dependent diabetes mellitus)) |
| 2 | Health coaching | ((health coach*) OR (health belief) OR (health behaviour) OR (health manage*) OR (health educat*) OR (health promot*) OR (health educat*) OR (wellness coach*) OR (lifestyle coach*) OR (nurse manage*) OR (case manage*) OR (prevention manage*) OR (prevention coach*) OR (motivational interview*)) |
| 3 | Remote management | (((Telemedicine) OR (tele-medicine) OR (telehealth) OR (tele-health) OR (Telemonitor*) OR (tele-monitor*) OR (ehealth) OR (electronic health) OR (e-health) OR (mobile health) OR (remote manag*) OR (remote monitor*) OR (remote consult*) OR (mhealth) or (m-health) OR (digital health) OR (Telematic) OR (tele-matic) OR (telemanag*) OR (Tele-manag*) OR (tele-nurs*) OR (telenurs*) OR (teleservic*) OR (tele-servic*) OR (tele-care) OR (telecare) OR (Tele-home) OR (telehome) OR (Tele-conferenc*) OR (Teleconferenc*) OR (Teleconsult*) OR (Tele-consult*) OR (telecommunicat*) OR (tele-communicat*))) |
