# Supplementary material for: Health Coaching and Its Impact in the Remote Management of Patients With Type 2 Diabetes Mellitus: Scoping Review of the Literature
Source: J Med Internet Res. 2025 Apr 9;27:e60703. doi: 10.2196/60703 (PMC12018868; doi:10.2196/60703)
Supplement: Multimedia Appendix 3 [file jmir_v27i1e60703_app3.docx]

**Multimedia Appendix 3.** Inclusion and exclusion criteria using the PICOS (patient, intervention, comparator, outcomes, and study) framework.

| Criteria | **Inclusion criteria** | **Exclusion criteria** |
| --- | --- | --- |
| Patient | - All adult Type 2 diabetes mellitus (T2DM) patients (Aged ≥ 18 years old)   - Regardless of T2DM duration   - On any form of remote monitoring (refer to below for definition)   **Remote monitoring**   - Defined as use of digital technology to capture patient’s health information in real-time and thereafter transmitted for evaluation by a healthcare professional or for self-management.   **Forms of remote monitoring included**   - Teleconsultation / video conferencing / telephones call for consultation or transmission of patient related outcomes - Use of automated devices such as wearable technologies (e.g. fitness watch), blood pressure devices and glucometer with transmission capability innately or via online web platforms / any forms of communication networks - Use of electronic devices e.g. blood pressure devices and glucometer with transmission capability innately or via online web platforms / any forms of communication networks - Use of online web or mobile platforms, electronic devices e.g. handphone / tablet or software for transmission of patient information and clinical information | Patient population   - Type 1 diabetes mellitus patients - Maturity onset diabetes of the young patients - Patients aged less than 18 years old   Patient who are not on any form of remote monitoring |
| Intervention | **Health coaching**   - health coaching was defined as “the practice of health education and health promotion within a coaching context to enhance the well-being of individuals and to facilitate achievement of their health-related goals” - that delivered via telephone, internet, online teleconferences, in person or usage of a combination of multiple delivery methods - No restrictions on types of health coaches | - Studies which did not perform health coaching or conduct remote monitoring of patients |
| Comparator | - Usual care - For single arm studies / trials: no comparator was considered acceptable and included |  |
| Outcomes | Types of outcomes   1. Clinical outcomes such as diabetes control, body mass index, blood pressure control, lipid control, renal outcomes, cardiovascular diseases, mortality, and morbidity 2. Humanistic outcomes such as health related quality of life, diabetes related distress 3. Psychiatric outcomes such as depression, anxiety, development of psychiatrics diseases 4. Behavioural outcomes such as adherence to dietary and exercise recommendations, medication adherence 5. Knowledge related outcomes such as diabetes related knowledge 6. Economic outcomes such as healthcare utilization, number of clinic visits, visits to accident and emergency department, hospitalisation, healthcare related costs | Studies which did not evaluate any outcomes |
| Study designs | - Randomized controlled trials - Cross-sectional studies - Cohort studies - Observational studies - Qualitative studies - Quasi-experimental - Mixed-methods studies | - Case reports or case series - Study protocols - Irrelevant systematic reviews or meta-analyses |
